# Revealing the Patterns and Key Genes of Heat-Stress Response in *Quinoa* Through Physiological and Transcriptomic Analyses

**DOI:** 10.3390/biology15191781

**Published:** 2026-10-08

**Authors:** Wenli Liu, Fangjun Chen, Wei Wang, Jiancheng Song, Shihua Chen, Yibo An, Kun Chen, Haibo Yin, Shanli Guo

**Affiliations:** 1 College of Life Sciences, Yantai University, Yantai 264005, China; 2 Shandong Laboratory of Advanced Agriculture Sciences at Weifang, Peking University Institute of Advanced Agricultural Sciences, Weifang 261325, China; 3 Chongqing Forestry Investment Development Co., Ltd., Chongqing 401120, China; 4 College of Life Science, Qilu Normal University, Jinan 250200, China

**Keywords:** quinoa, heat stress, transcriptome, physiology, WGCNA

## Abstract

Quinoa is a high-nutrition crop originating from the Andes Mountains that is tolerant to cold, drought and salt. However, the mechanism of the high-temperature response in quinoa is still unclear. In this study, quinoa line YL-6 was used to analyze the changes in photosynthetic parameters, physiological parameters and the transcriptome after 0, 3, 12 and 24 h of heat stress. Our results showed that photosynthetic parameters, including the maximum quantum yield of PSII (Fv/Fm), steady-state effective quantum yield (ΦPSII_Lss), non-photochemical quenching (NPQ_Lss), and photochemical quenching coefficients (qL_Lss, qP_Lss), decreased significantly under heat stress, whereas the activities of antioxidant enzymes, including superoxide dismutase (SOD), peroxidase (POD), and catalase (CAT), increased significantly at the early stage. The contents of osmotic adjustment substances such as proline, soluble protein and soluble sugar showed a non-single change trend. WGCNA was conducted to group the differentially expressed genes (DEGs) into 12 co-expression modules. Key modules associated with heat perception and signal transduction were identified, and a core regulatory network was subsequently constructed. Hub genes encoding molecular chaperones and transcription factors were screened, among which heat shock proteins (HSPs) were the most prominent. These results could provide a theoretical basis for analyzing the regulatory network of heat stress, exploring heat-resistant genes and introducing and domesticating quinoa.

## Simple Summary

Quinoa (*Chenopodium quinoa* Willd.) is tolerant to cold, drought and salt, whereas the molecular mechanism underlying its response to high temperature remains poorly understood. In this study, quinoa line YL-6 was used to determine photosynthetic and physiological parameters and perform transcriptome analysis under heat stress for different durations. High temperature markedly suppressed photosynthesis and increased the activities of antioxidant enzymes at the early stress stage, while osmotic adjustment substances exhibited a complex changing pattern. Weighted gene co-expression network analysis (WGCNA) identified key modules associated with heat signaling and hub genes dominated by heat-shock proteins. This study provides a theoretical basis for dissecting the thermotolerance regulatory network, exploring heat-tolerant genes, and supporting the introduction and domestication of quinoa.

## 1. Introduction

*Chenopodium quinoa* Willd., commonly known as quinoa, is an annual dicotyledonous species belonging to the Chenopodiaceae family, with a domestication and cultivation history spanning approximately seven millennia across the Andean highlands, including Bolivia, Chile, Argentina, Colombia, and Peru [1,2]. Compared with conventional cereal crops, quinoa grains are distinguished by their exceptional nutritional profile, boasting abundant essential amino acids, dietary fiber, lipids, carbohydrates, vitamins, and minerals [3]. The Food and Agriculture Organization of the United Nations (FAO) has identified quinoa as the only standalone crop capable of fulfilling all essential nutritional demands for human beings. This crop has therefore attracted wide international attention. Quinoa can resist most abiotic stresses, such as high salinity, drought and low temperature, so that it can adapt to more variable climatic regions [4]. In a high-temperature environment, the tolerance of quinoa decreases significantly. While the germination rate of seeds may remain relatively high in the germination stage, the stomatal conductance, photosynthesis, chlorophyll fluorescence and chlorophyll content of quinoa leaves decrease to varying degrees in the vegetative developmental stage and reproductive developmental stage. As these phenomena seriously affect the normal growth and seed yield in quinoa, improving tolerance to heat and biological stresses is considered the main breeding objective of quinoa genetic improvement, apart from breeding more compact and dwarf lines [5].

High-temperature stress (HS) is one of the common abiotic stresses in the process of plant growth and development, and it usually disrupts the normal metabolism of plants by degrading proteins, resulting in protein misfolding and loss of biological functions, and further causing growth and development retardation [6]. The types of damage caused by high-temperature stress can be divided into direct damage and indirect damage. The former leads to cell structure rupture and tissue necrosis, while the latter destroys the biofilm system and affects the normal metabolic rate [7]. Without irrigation, heat stress can improve the carbon assimilation capacity of leaves within certain limits, but it will increase the water demand of plants, which will lead to water stress and yield decline of plants [8]. Therefore, it is necessary to further understand the physiological responses of plants to high-temperature stress, the mechanism of heat tolerance and possible strategies to improve the heat tolerance of crops. The heat shock response affects the developmental process of the overall growth and development of plants, although the thermal threshold level varies greatly at different developmental stages and in different species. For example, during seed germination, high temperatures may slow or completely inhibit germination, depending on the plant species and the intensity of the stress [9]. In later stages, high temperatures adversely affect photosynthesis, respiration, water relations, and membrane stability and also regulate the levels of hormones and primary and secondary metabolites [10]. Furthermore, the synthesis of diverse HSPs and other stress-associated proteins, alongside reactive oxygen species (ROS), represents the core defensive response network that plants deploy against high-temperature stress across their entire life cycle. When exposed to heat stress, plants activate a multifaceted set of protective mechanisms. These include preserving membrane structural integrity, scavenging excess reactive oxygen species, synthesizing antioxidant compounds, accumulating osmoprotective solutes, triggering mitogen-activated protein kinase (MAPK) and calcium-dependent protein kinase (CDPK) signaling cascades, and upregulating the expression of stress-responsive genes and proteins. Among these, HSPs serve as key molecular chaperones that prevent the aggregation of misfolded proteins and assist in the refolding of heat-denatured proteins, thereby maintaining cellular homeostasis and enhancing plant thermotolerance [11]. The most important of these are the transduction and transcriptional activation of chaperone signals. All these measures can alleviate heat stress to a certain extent at the molecular level.

Plant response pathways to high-temperature stress can be divided into the following four categories: heat shock transcription factor–heat shock protein (HSF-HSP) pathway, Ca^2+^–calmodulin (Ca^2+^-CaM) pathway, reactive oxygen species (ROS) pathway and hormone pathway [12]. Among them, the HSF-HSP pathway is the main pathway that plays an important role in the process of plant resistance to high temperature [12]. Under high-temperature stress, the specific Ca^2+^ channel on the cell plasma membrane will open, leading to Ca^2+^ influx, followed by the activation of multiple Ca^2+^-dependent protein kinases (calcium-dependent protein kinases, CDPKs) and calcium/calmodulin-binding protein kinase (CBK), thus initiating the expression of downstream genes related to high-temperature stress [10,13]. Under high-temperature conditions, plant cells undergo a burst of reactive oxygen species (ROS), leading to severe oxidative stress. Subsequently, this oxidative cue transduces heat-related signaling molecules into the nuclear compartment, where they bind to specific cis-elements within the promoters of heat shock genes. This binding event subsequently triggers the transcriptional activation of heat shock genes, thereby effectively mitigating the heat-induced cellular damage in plants [14]. At the same time, plant hormones such as salicylic acid (SA), abscisic acid (ABA) and ethylene are also closely related to heat shock signal transduction [15]. Within the HSF-HSP signaling cascade, heat shock factors (HSFs) act as the terminal effectors of the heat-stress signal transduction pathway. Upon perceiving heat shock signals, HSFs directly modulate the expression of downstream heat-stress responsive genes, with HSPs representing the primary and most critical targets of HSF-mediated regulation. Collectively, the HSF-HSP module constitutes a well-characterized, classical heat-stress response pathway that plays a central role in plant thermotolerance and adaptation to high-temperature stress [16]. In addition to HSFs, there are transcription factors related to heat stress, such as PIF and WRKY, which play a certain regulatory role in the response to heat stress [17].

The growth and development of quinoa are generally inhibited under heat stress, and it is very important to explore the mechanism of the heat-stress response in quinoa, but the understanding of this mechanism is still very limited. In this study, we compared the transcriptomic profiles of quinoa line YL-6 under four different heat treatment durations (0 h, 3 h, 12 h, and 24 h) to explore the molecular changes in heat-stress response and to identify key genes in signal transduction pathways. Combining physiological, biochemical and transcriptomic data, we summarized the main processes of quinoa in response to heat stress and screened out some candidate genes that could enhance the early heat-stress response (HSR) without adverse growth-related effects. This study illustrated the response of quinoa to heat stress at the physiological and molecular levels, which could provide valuable molecular references for investigating other quinoa varieties and *Chenopodium* species.

## 2. Materials and Methods

### 2.1. Plant Material and Treatment

*Chenopodium quinoa* cultivar YL-6 used in this study was preserved and provided by our research laboratory. Seedlings were cultivated in the plant improvement nursery of Yantai University (121°46′ E, 37°58′ N, Shandong Province, China). Plump seeds with uniform size were planted in small square pots filled with a sterilized substrate (vermiculite:Pinkerton peat soil, 3:1) and placed in a growth room with an ambient temperature of 24 °C and a photoperiod of 16 h light/8 h darkness for germination. When seedlings had developed to the stage of six expanded leaves and one heart, seedlings in the treatment group were transferred to greenhouses maintained at a constant temperature of 40 °C, while the control group remained at 24 °C. Sampling was carried out after heat treatment for 3 h, 12 h and 24 h. Leaves (the fourth or fifth leaf) at identical positions were harvested from three different plants and pooled to form one biological sample. The control group was also sampled in parallel. For physiological index detection, samples were used immediately after harvest, whereas sequencing samples were frozen for 5–10 min in liquid nitrogen immediately after harvest.

### 2.2. Chlorophyll Fluorescence Imaging System

Chlorophyll fluorescence was measured using an IMAGING-PAM chlorophyll fluorometer (Walz GmbH, Effeltrich, Germany) with ImagingWin software (version 2.47). The third and fourth fully expanded leaves from the apex were detached and immediately dark-adapted for 30 min. The minimum (F0) and maximum (Fm) fluorescence were recorded, and the maximum quantum yield of PSII was calculated as Fv/Fm = (Fm − F0)/Fm. The leaves were then exposed to actinic light at 300 µmol m^−2^ s^−1^ (calibrated with a quantum sensor), during which saturating pulses (approximately 4000 µmol m^−2^ s^−1^, 800 ms) were applied every 20 s until steady-state fluorescence was reached. From the steady-state fluorescence (F), light-adapted maximum fluorescence (Fm′) and light-adapted minimum fluorescence (F0′ = F0/(Fv/Fm + F0/Fm′)), the following light-adapted steady-state parameters were calculated: the effective quantum yield of PSII, ΦPSII_Lss = (Fm′ − F)/Fm′; non-photochemical quenching, NPQ_Lss = (Fm − Fm′)/Fm′; photochemical quenching, qP_Lss = (Fm′ − F)/(Fm′ − F0′); the fraction of open ΦPSII reaction centers, qL_Lss = qP × F0′/F; and the non-photochemical quenching coefficient, qN_Lss = 1 − (Fm′ − F0′)/(Fm − F0). It should be noted that the measurement points were positioned away from the main leaf vein [18].

### 2.3. Detection of Biochemical Indexes of Quinoa Leaves Under Heat Stress

Superoxide dismutase (SOD) activity was determined following the protocol described by Giannopolitis and Ries [19]. Briefly, leaf samples were homogenized in pre-cooled phosphate buffer for crude enzyme extraction, and nitroblue tetrazolium (NBT) was used as the reaction substrate. Peroxidase (POD) activity was assessed according to the method reported by Scebba et al. [20]. Enzymes were extracted using phosphate buffer, and guaiacol served as the substrate for the POD reaction. Catalase (CAT) activity was detected using the modified approach established by Chance and Maehly [21]. After enzyme extraction in phosphate buffer, hydrogen peroxide was used as the substrate for the CAT catalytic reaction. The proline content (Pro) was determined using the acid-ninhydrin method [22]. In brief, leaf samples were extracted with 3% sulfosalicylic acid. Under acidic boiling conditions, proline specifically forms a stable red chromophore with ninhydrin to reduce interference from other amino acids. The absorbance was determined at 520 nm for quantification. The soluble protein (SP) content was quantified using the Coomassie brilliant blue G-250 staining method [23], based on protein binding to Coomassie dye to produce a blue complex. The soluble sugar content (SS) was determined using the anthrone colorimetric method [24], in which carbohydrates react with anthrone under heating to form a colored product. All measurements were performed with three biological replicates.

### 2.4. RNA Extraction, cDNA Library Construction and Illumina Sequencing

Total RNA was extracted from the samples harvested at different time points, and mRNA was purified from the total RNA with oligonucleotide (dT) beads for library preparation. A total of 12 cDNA libraries were constructed from the mRNA of 3 independent biological samples at 4 time points; the purity of the RNA was determined by a NanoDrop 2000 ultra-micro ultraviolet spectrophotometer (Thermo Fisher Scientific, Waltham, MA, USA), and the integrity of the RNA was determined by an Agilent 2100/LabChip GX (PerkinElmer, Waltham, MA, USA). The library was sequenced in PE150 mode using the Illumina NovaSeq 6000 sequencing platform (Illumina, San Diego, CA, USA).

### 2.5. RNA-Seq Data Quality Control, Alignment and Assembly

Clean data were obtained by removing primers, adapter sequences and low-quality raw reads with ambiguous base N (including reads with N removed more than 10% and reads in which bases with Q ≤ 10 accounted for more than 50% of the total read length). HISAT2 software (version 2.2.1) [25] was used to align clean reads against the reference genome of quinoa (ASM168347v1) [26], and the location information of reads on the reference genome was retrieved to generate mapped data. The aligned reads were then assembled using StringTie for subsequent analysis [27].

### 2.6. Identification of Differentially Expressed Genes (DEGs)

First, FPKM (Fragments Per Kilobase of transcripts per Million fragments mapped) was used for normalization as an index to measure the expression level of transcripts or genes [28]. The difference analysis was then performed using the DESeq2 software (version 1.42.0) [29]. Differentially expressed genes were identified using the thresholds of |fold change| ≥ 2 and FDR < 0.05. Here, fold change refers to the ratio of transcript abundance between the two comparison groups.

### 2.7. Construction of Weighted Gene Co-Expression Networks Analysis (WGCNA)

WGCNA is a powerful tool for constructing RNA-Seq gene co-expression networks [30]. Co-regulated genes with potentially similar biological functions can be effectively identified by this method. We used WGCNA (version 1.73) to construct a co-expression network using the first 6000 genes with median absolute deviation of FPKM values as target genes for the analysis [30]. Gene expression pattern clustering analysis was performed using Mfuzz (version 2.68.0) [31], and the resulting co-expression network was visualized using Cytoscape (version 3.9.1) [32].

### 2.8. GO and KEGG Enrichment Analysis of Differentially Expressed Genes

In order to understand the potential biological functions of DEGs, we used the clus- terProfiler R package to map DEGs to the GO (http://www.geneontology.org/, accessed on 6 July 2025) and KEGG (http://www.genome.jp/kegg/, accessed on 6 July 2025) databases [33,34,35]. GO or KEGG terms with *p*-values < 0.05 were considered to be significantly enriched in DEGs.

### 2.9. Transcription Factor (TF) Prediction for DEGs

For TF prediction, tools on the Plant Transcription Factor Database v4.0 (http://planttfdb.cbi.pku.edu.cn, accessed on 7 August 2025) were used [36]. Coding regions within the nucleic acid sequences were identified and translated using ESTScan (version 3.0) [37].

### 2.10. qRT-PCR Validation

Parallel samples from RNA-Seq were used for qRT-PCR detection. Eight DEGs were randomly selected, and Primer Premier 5.0 software was used to design specific primers for the selected gene sequences, which were aligned on the BLAST tool (https://blast.ncbi.nlm.nih.gov/Blast.cgi, accessed on 27 August 2025) at NCBI and synthesized by Shenzhen BGI Co., Ltd. (Shenzhen, China). Quinoa *tubulin* was used as the reference gene. Real-time quantitative reverse transcription polymerase chain reaction (qRT-PCR) was performed using a LightCycler 480 Real-Time PCR System (Roche Diagnostics, Mannheim, Germany) and quantitative variation was assessed using a relative quantitation method (2^−ΔΔCt^). The RNA samples used for quantitative qRT-PCR were identical to those used for Illumina sequencing.

### 2.11. Statistical Analysis

In this study, three biological replicates of the same sampling time point were analyzed, and the data are presented as the mean ± SD. One-way analysis of variance (ANOVA) and Student’s *t*-test were used to assess statistical significance.

## 3. Results

### 3.1. Chlorophyll Fluorescence Imaging Reveals the Response of Quinoa Leaves to Heat Stress

**Figure 1:**
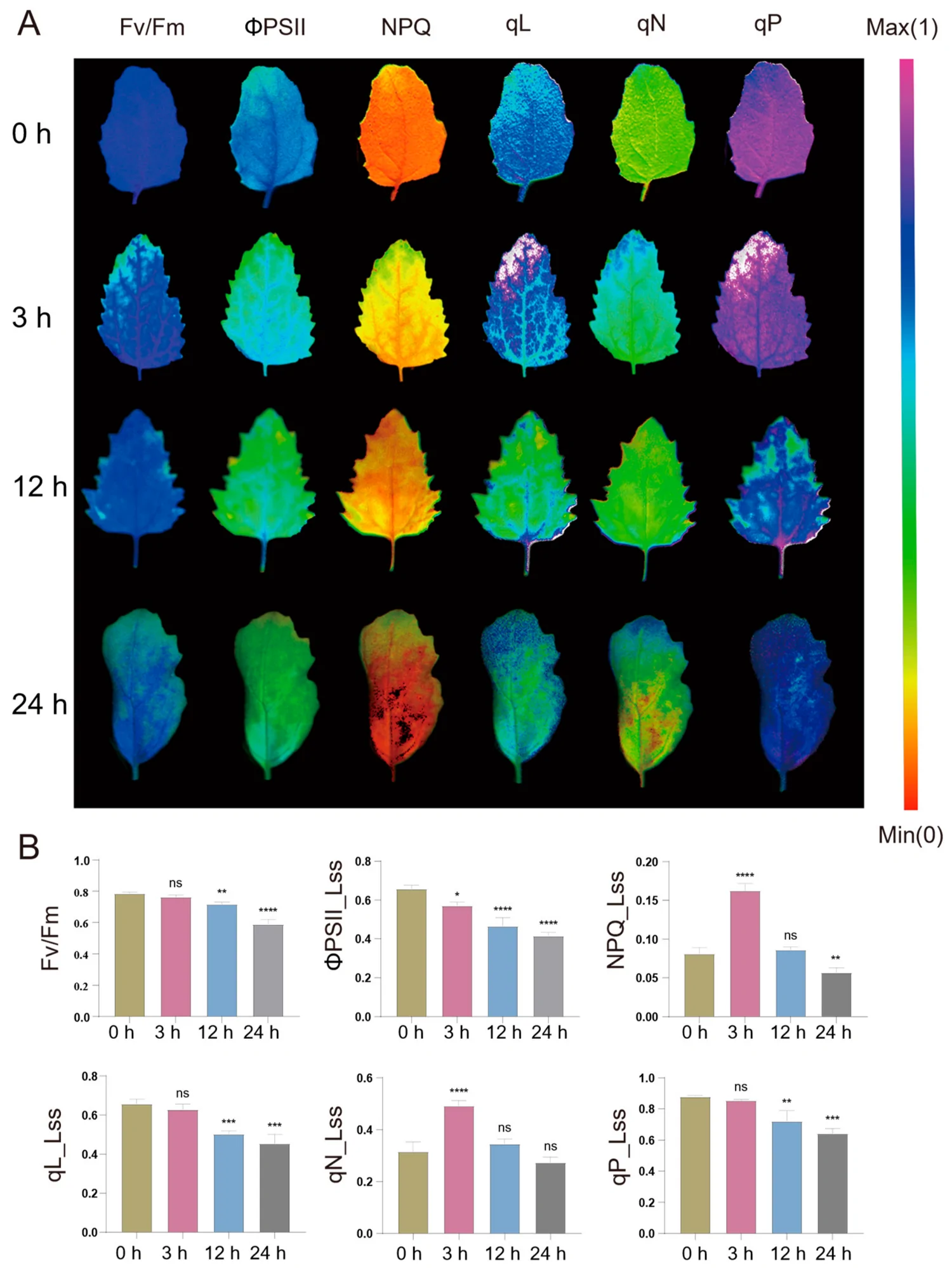
Analysis of chlorophyll fluorescence and related photosynthetic parameters of quinoa (*Chenopodium quinoa* Willd.) leaves at different heat-stress time points. (**A**) Images of chlorophyll fluorescence parameters showing changes in Fv/Fm (maximum photochemical efficiency of PSII), ΦPSII_Lss (actual photochemical efficiency of photosystem II), NPQ_Lss (non-photochemical quenching), qL_Lss (photochemical quenching coefficient), qN_Lss (non-photochemical quenching coefficient), and qP_Lss (photochemical quenching) at different heat-stress time points. The color scale represents parameter values from minimum (Min, red) to maximum (Max, purple), with numerical values indicated on the scale. (**B**) Histograms of each photosynthetic parameter, showing the statistical differences of Fv/Fm, PSII_Lss, NPQ_Lss, qL_Lss, qN_Lss and qP_Lss at different time points, with statistically significant differences indicated (ns *p* > 0.05, * *p* ≤ 0.05, ** *p* ≤ 0.01, *** *p* ≤ 0.001, **** *p* ≤ 0.0001). Data are presented as mean ± SD (*n* = 3).

As shown in Figure 1, we measured the photosynthetic parameters of quinoa leaves under heat stress at 40 °C using an IMAGING-PAM chlorophyll fluorometer. It is used to visually display the dynamic effects of treatment time on the photosynthetic physiological state of the leaves. Fv/Fm fluorescence at 3 h vs. 0 h showed a significant change at the top edge of the leaf, but the maximum quantum efficiency of PSII (Fv/Fm) did not decrease significantly (4.3%), which may be due to the fact that there was no significant change near the main vein of the leaf; however, 12 h vs. 0 h showed a significant decrease (9.1%), and Fv/Fm decreased continuously (25.4%) under heat stress. ΦPSII_Lss and Fv/Fm showed a similar trend over time, but the difference was that ΦPSII_Lss had a significant decrease (13.2%) compared with Fv/Fm at 3 h vs. 0 h. The NPQ increased sharply (100%) at 3 h vs. 0 h and had no significant change (ns) at 12 h vs. 0 h but did decrease significantly (30.1%) at 24 h vs. 0 h, indicating that the photoprotective mechanism was enhanced in the early stage and that the photoprotective ability was weakened in the later stage. The qL_Lss did not change significantly at 3 h vs. 0 h (ns) but decreased significantly at the later stages of 12 h and 24 h (23.5% and 30.1%), and the open proportion of PSII reaction centers decreased with time. qN_Lss increased significantly (56.3%) at 3 h vs. 0 h but did not change significantly at 12 h and 24 h compared with 0 h (ns), indicating that non-photochemical quenching was enhanced in the early stage and remained stable in the later stage. The qP_Lss did not change significantly at 3 h vs. 0 h (ns) but decreased significantly at the later stages of 12 h and 24 h (18.9% and 26.9%) compared with 0 h, indicating that the potential opening capacity of the PSII reaction centers decreased with time. It can be seen that the photochemical efficiency of PSII (Fv/Fm, ΦPSII, qL and qP) generally decreased with time (0 h → 24 h), while the non-photochemical quenching (NPQ_Lss and qN_Lss) increased significantly in the early stage (0 h → 3 h), and some photoprotective indicators (such as NPQ_Lss) decreased in the later stage (12 h → 24 h). These observations reflected that the function of PSII was gradually impaired with the extension of treatment time.

### 3.2. Changes in Osmotic Adjustment Substances and Antioxidant Enzyme Activities in Quinoa Leaves at Different Heat-Stress Time Points

**Figure 2:**
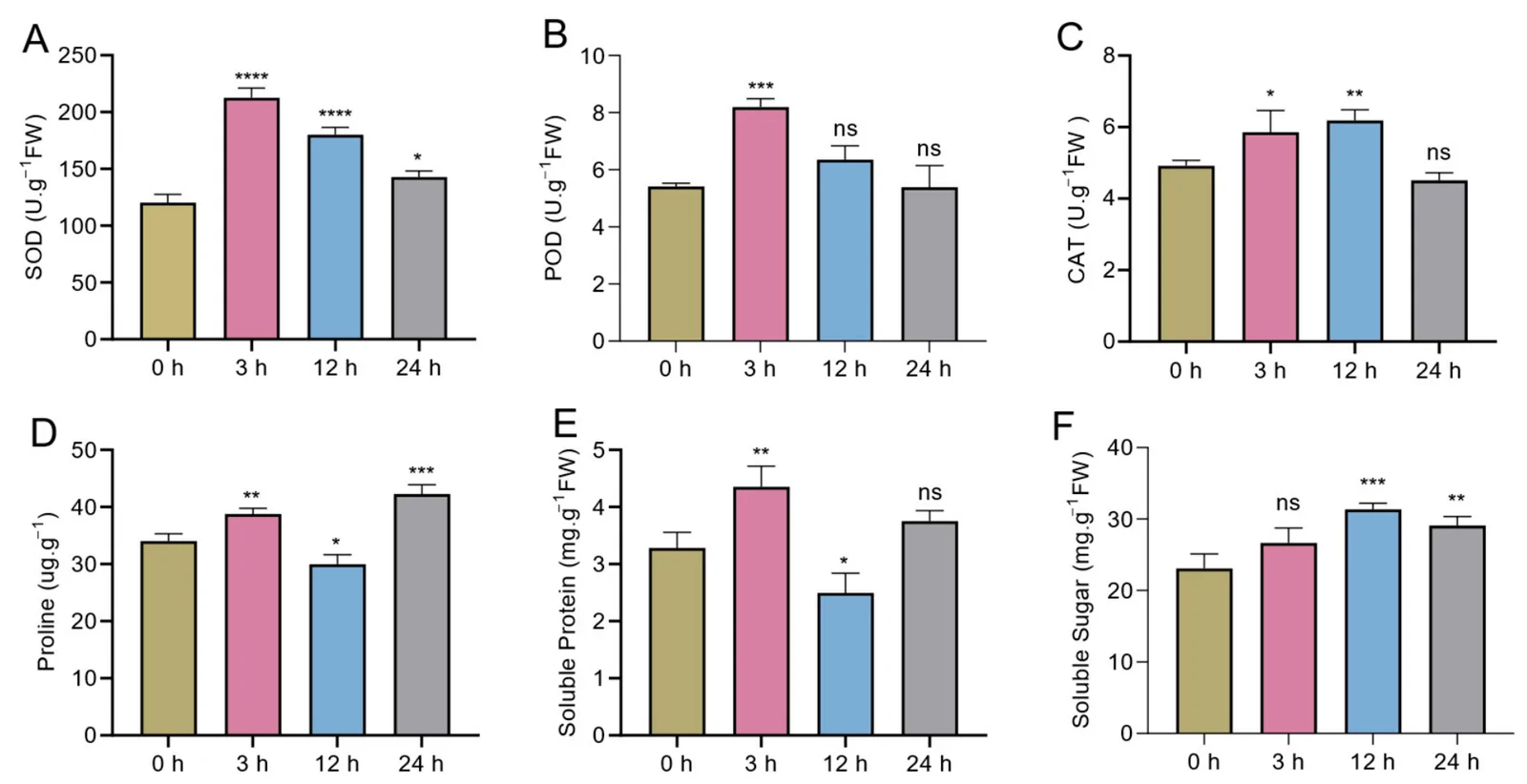
Variations in antioxidant enzyme activities and osmolyte contents in quinoa leaves under heat stress at different time points. Assays included: (**A**) superoxide dismutase (SOD), (**B**) peroxidase (POD), and (**C**) catalase (CAT) activities, as well as (**D**) proline (Pro), (**E**) soluble protein (SP), and (**F**) soluble sugar (SS) contents. Significance levels are marked as follows: ns *p* > 0.05, * *p* ≤ 0.05, ** *p* ≤ 0.01, *** *p* ≤ 0.001, **** *p* ≤ 0.0001. This figure illustrates the dynamic changes in plant antioxidant defense and osmotic adjustment systems under prolonged heat treatment. Data are presented as mean ± SD (*n* = 3).

As shown in Figure 2, heat stress significantly affected the activities of active oxygen scavenging enzymes in quinoa leaves. SOD activity increased significantly at 3 h vs. 0 h (76.7%), reaching its peak, and then declined at 12 h vs. 0 h (49.8%) and decreased slightly at 24 h vs. 0 h but was still significantly higher than CK (18.6%). Although the change trend of POD was similar to that of SOD, there was a significant difference (51.4%) only at 3 h vs. 0 h. CAT showed a different trend and reached a peak (25.7%) at 12 h vs. 0 h. In addition, heat stress also significantly affected the content of osmotic adjustment substances in quinoa leaves. The proline content increased significantly (14%) at 3 h vs. 0 h, then decreased significantly (11.9%) at 12 h vs. 0 h, and reached a peak (24.3%) at 24 h vs. 0 h. Soluble protein showed a significant increase (32.7%) at 3 h vs. 0 h, a significant decrease (24%) at 12 h vs. 0 h, and a non-significant increase (14.4%) at 24 h vs. 0 h. Soluble sugar exhibited a different change trend from proline and soluble protein. It increased at 3 h vs. 0 h (15.4%), but there was no significant difference; it then increased to a peak at 12 h vs. 0 h (35.9%) and decreased at 24 h vs. 12 h. However, there was still a significant increase (25.9%) at 24 h vs. 0 h.

### 3.3. High-Throughput Transcriptome Sequencing and Sequence Assembly

The main characteristics of the 12 cDNA libraries consisting of three independent biological samples from four treatment groups (0, 3, 12, and 24 h) are shown in Table 1. The 12 libraries generated a total of 514,977,962 raw reads using Illumina sequencing, with an average of 42,914,830 reads per sample. After removal of linker sequences, ambiguous nucleotides, and low-quality reads, 12 libraries yielded 19,483,485 to 27,480,003 clean reads, with an average of 21,457,415 clean reads per sample. The Q20 and Q30 values of the clean data were over 96% and 91%, respectively, indicating that the sequencing data were good and reliable. The percentage of G+C was about 43%. At the same time, we found that more than 96% of the clean reads were mapped to the reference genome, of which more than 88% can be uniquely mapped to the reference genome, with an average mapping rate of 89.37%. Sequences encoding short peptides (less than 50 amino acid residues) or containing only a single exon were filtered out, and a total of 8594 novel transcripts were identified (Tables S1 and S2). The correlation between the biological replicates was above 0.962, indicating that the quality of the samples was good and that the error of the repeated samples was small, so the sequencing results were reliable (Figure S1). The twelve transcriptome datasets have been deposited in the NCBI Sequence Read Archive (SRA) under accession number PRJNA1446775.

### 3.4. Gene Expression Under Different Heat-Stress Conditions

**Figure 3:**
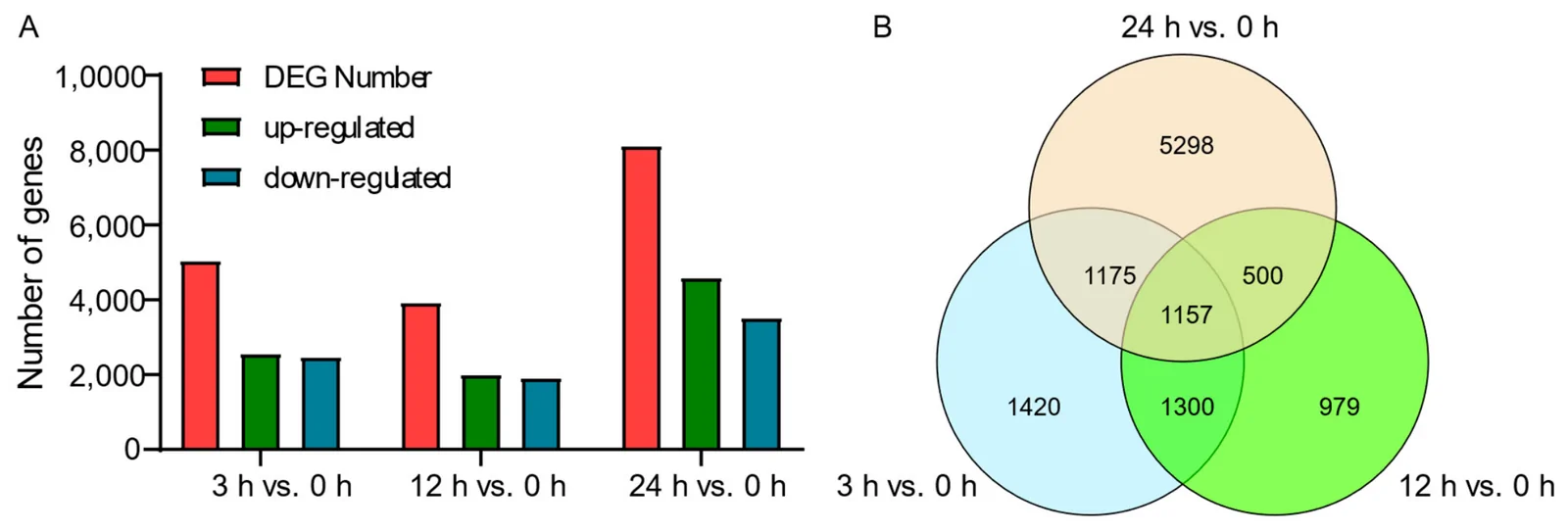
Gene expression patterns and cluster heatmaps of quinoa under different durations of heat stress. (**A**) Numbers of up- and down-regulated genes at each time point relative to the control. Red and green bars indicate up- and down-regulated genes, respectively. (**B**) Venn diagram showing the distribution of differentially expressed genes among three heat treatment groups versus the control.

Differential analysis of gene expression was performed with samples under 40 °C for three different time points (3, 12, and 24 h) compared with the control group under 24 °C. From 0 h to 3 h, 5052 differentially expressed genes were identified, of which 2481 genes were down-regulated, 2571 genes were up-regulated, and the number of up-regulated and down-regulated genes was almost the same. From 0 h to 12 h, 3936 differentially expressed genes were identified, of which 1923 genes were down-regulated, and 2013 genes were up-regulated, with the number of up-regulated and down-regulated genes being almost the same. From 0 h to 24 h, there were 8130 differentially expressed genes, of which 3530 genes were down-regulated, and 4600 genes were up-regulated, with significantly more up-regulated genes than down-regulated genes (Figure 3A). The above results showed that most of the genes related to heat stress exhibited a delayed-type response pattern, and these genes reached their expression peak at 24 h versus 0 h. The above results showed that the expression peak was reached in the comparison between 0 h and 24 h, indicating that most of the heat-stress-related genes exhibited a delayed-type response, which indicated that the gene set was of great significance for quinoa to cope with heat stress (Figure 3B).

### 3.5. Gene Clusters and Expression Pattern Analysis at Different Heat-Stress Time Points

**Figure 4:**
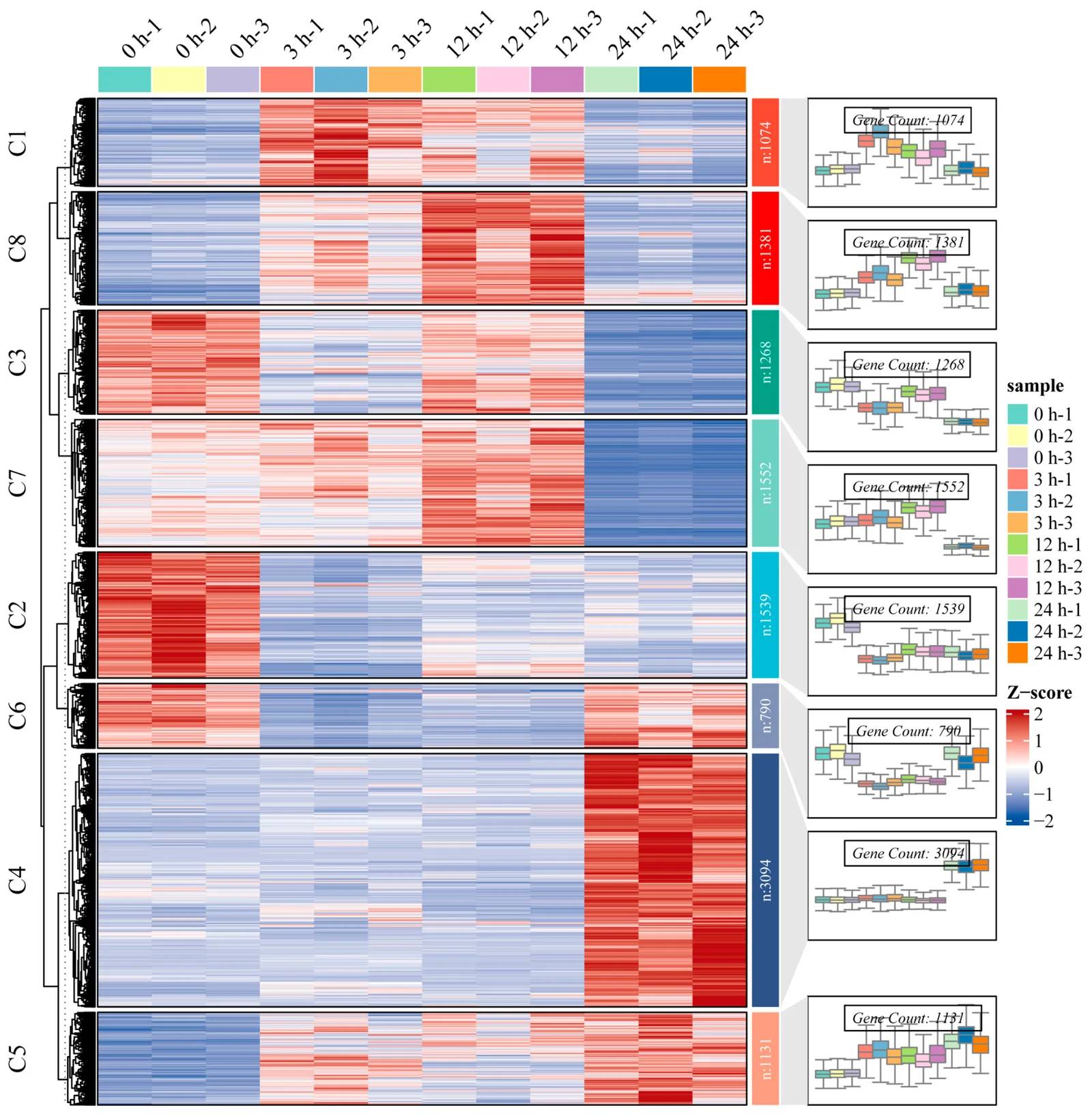
Gene expression clustering heatmap and clustering analysis based on Z-score normalization. The left side are the clusters of different genes (C1–C8), and the right side of each cluster is marked with the number of genes; the abscissa shows the samples, and the color coding corresponds to the legend; the color shade (red–blue gradient) represents the Z-score (−2 to 2) of gene expression, reflecting the degree of deviation of the expression level from the mean. The sub-graph on the right side is the hierarchical clustering tree of samples in each gene cluster, and the gene count of the corresponding cluster is marked to show the clustering characteristics of gene expression patterns and the structural differences of sub-clusters under different sample groupings.

As shown in Figure 4, DEGs were clustered into eight expression clusters using the Mfuzz package based on Z-score normalized expression profiles, and all eight clusters were retained for further analysis. There were significant clustering and expression patterns of genes during heat-stress treatment, which could be roughly divided into three main response expression patterns. Type I includes genes that are significantly down-regulated at all three heat-stress time points, which are mainly gathered in Cluster 2 and Cluster 3. Type II includes genes that are significantly up-regulated after 3 h to 12 h of heat stress, mainly grouped in Cluster 1, Cluster 4, Cluster 5 and Cluster 8, of which Cluster 1 and Cluster 8 are significantly up-regulated mainly at 3 h and 12 h. Cluster 4 is significantly up-regulated mainly at 24 h, and Cluster 4 contains the most DEGs, while Cluster 5 is significantly up-regulated at all time points. Type III represents the gene set that is significantly down-regulated at specific time points, mainly including Cluster 6 and Cluster 7. Cluster 6 is significantly down-regulated at 3 h and 12 h and basically recovers to the 0 h expression level at 24 h, and it is the gene set with the fewest DEGs. Cluster 7 represents a gene set in which differential expression changes are not obvious at 3 h and 12 h, while it is significantly down-regulated at 24 h. These can also be seen from the cluster trend representation boxplot. The expression profile of DEGs showed that the early regulated genes are mainly gathered in Cluster 1 at 3 h, and the middle regulated genes are mainly grouped in Cluster 7 and Cluster 8 at 12 h. In summary, 24 h may represent a critical transition point at which key genes and transcriptional regulators are engaged to initiate heat-responsive gene expression and signal transduction, thereby establishing a coordinated stress response framework.

### 3.6. Dynamic Changes in Transcription Factors During Heat Stress

**Figure 5:**
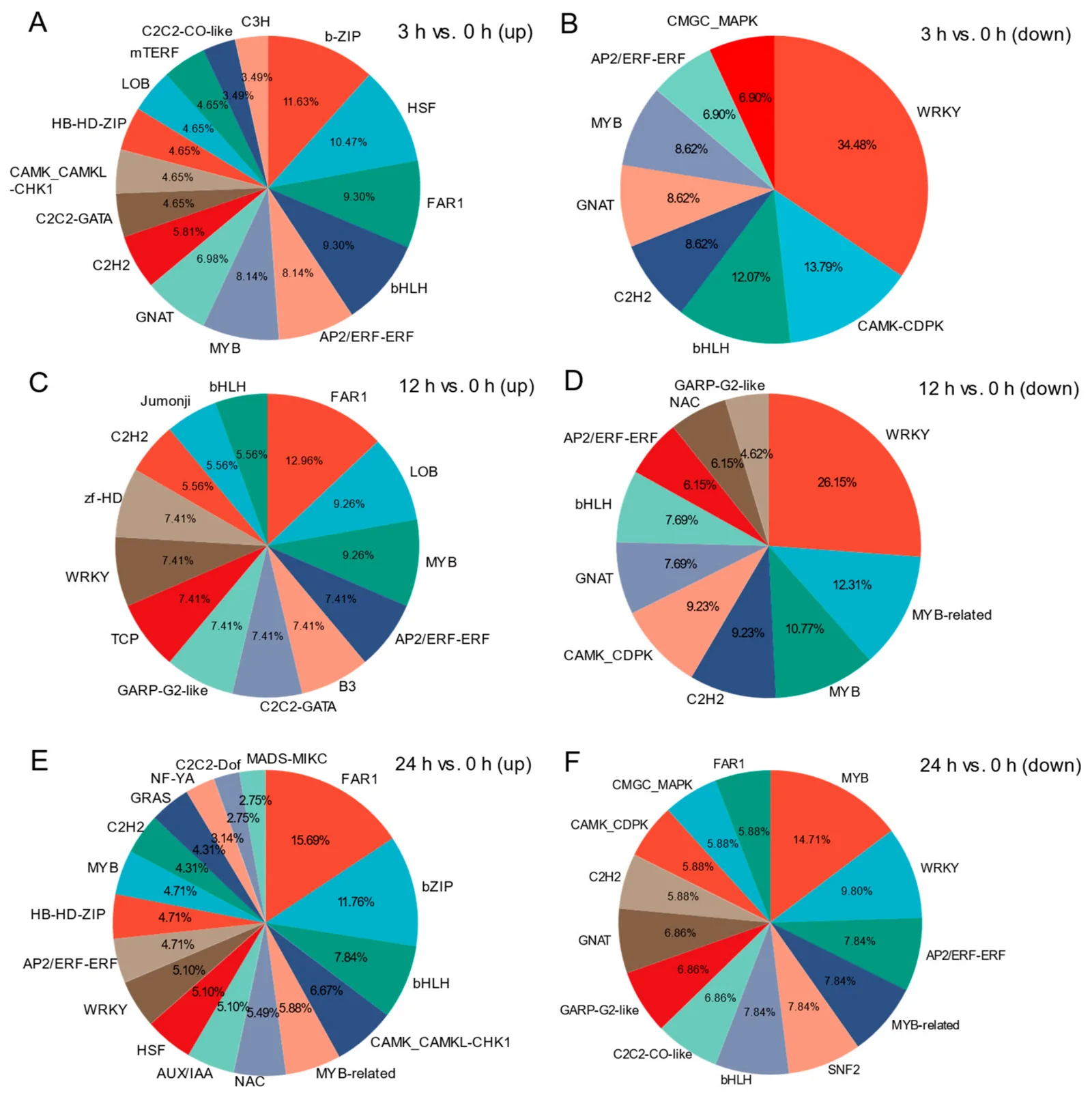
Composition of transcription factors in up-regulated and down-regulated differentially expressed genes (DEGs) in quinoa at different stages of heat stress. (**A**) 3 h vs. 0 h, up-regulated TFs (*n* = 86); (**B**) 3 h vs. 0 h, down-regulated TFs (*n* = 58); (**C**) 12 h vs. 0 h, up-regulated TFs (*n* = 54); (**D**) 12 h vs. 0 h, down-regulated TFs (*n* = 65); (**E**) 24 h vs. 0 h, up-regulated TFs (*n* = 262); (**F**) 24 h vs. 0 h, down-regulated TFs (*n* = 102). Each sector represents a transcription factor (TF) family, and the percentage value denotes the proportion of that family among all differentially expressed TFs in the corresponding group.

As shown in Figure 5, a total of 33 transcription factor families were identified among all DEGs (Table S3). For the DEGs from the 0 h vs. 3 h comparison group, 18 transcription factor families were annotated, 15 of which were up-regulated, involving 85 genes, with bZIP accounting for the largest proportion (11.63%). Eight transcription factor families were down-regulated, involving 58 genes, with WRKY accounting for the largest proportion (34.48%). Members that were both up-regulated and down-regulated are bHLH, C2H2, GNAT, MYB, and members of the AP2/ERF-ERF transcription factor family. For the DEGs in the control group comparison between 0 h and 12 h, 15 transcription factor families were annotated, nine of which were up-regulated, involving 65 genes, with FAR1 accounting for the largest proportion (12.96%). TF families containing 54 genes were down-regulated, among which the WRKY family accounted for the largest proportion (26.15%). Members that are both up-regulated and down-regulated are WRKY, C2H2, MYB, bHLH, and members of the GARP-G2-like transcription factor family. In the control group, six specific transcription factor families were only found in the down-regulated DEGs at 0 h vs. 24 h, with FAR1 accounting for the largest proportion (15.69%). Among the down-regulated transcription factor families, 10 specific transcription factor families were only found in the up-regulated DEGs, with MYB accounting for the largest proportion (14.71%). Seven transcription factor families, including MYB, WRKY, and bHLH, had both up-regulated and down-regulated genes.

### 3.7. WGCNA of Gene Expression Profiles and Module–Trait Relationships

**Figure 6:**
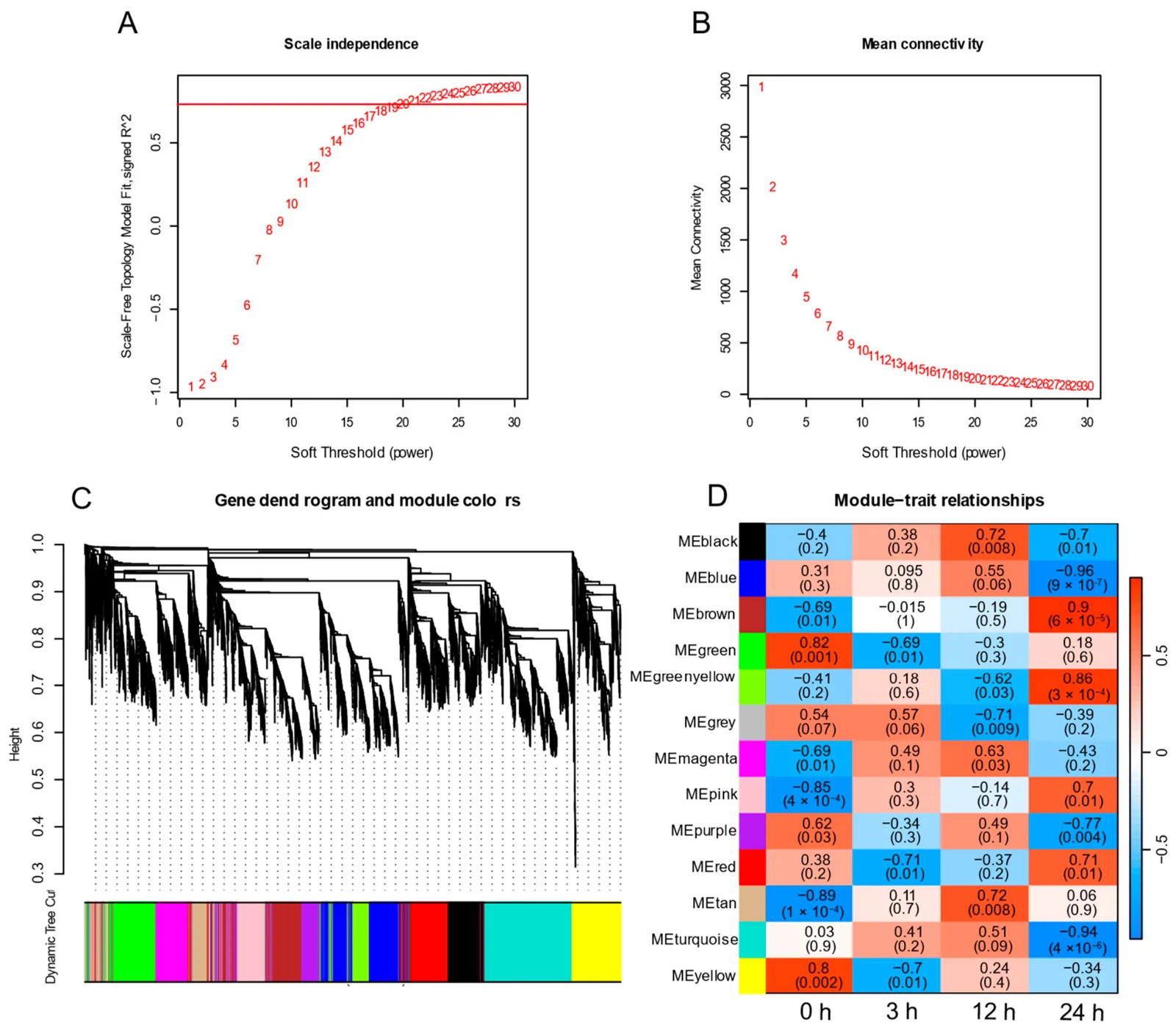
Determination of soft thresholds (β) and trait correlation analysis for WGCNA. (**A**) Determination of soft threshold, showing the relationship between soft threshold and the scale-free topology fitting index (R^2^), which was used to determine the optimal soft threshold for constructing the scale-free network; (**B**) presentation of the correlation between soft threshold and average connectivity; (**C**) gene-level cluster dendrogram (based on the dynamic tree-cut algorithm) and module color division, in which different colors represent different co-expression modules; (**D**) the heatmap shows the correlations between each module eigengene (ME) and four traits related to heat-stress treatment at different time points (0 h, 3 h, 12 h, 24 h). The color intensity (red–blue gradient) and numerical values represent Pearson correlation coefficients, and values in parentheses are raw *p*-values from Pearson correlation tests.

To reveal the underlying regulatory network during the early stage of heat stress in quinoa, we performed WGCNA of DEGs (Table S4). The appropriate soft-thresholding power (β) was determined using the pickSoftThreshold function. When β = 20, the scale-free network fitting index R^2^ was greater than 0.8, and the average connectivity approached 0. The scale-free network was constructed using this soft threshold (Figure 6A,B). When β = 20, 12 modules were generated (the number of genes ranged from 235 to 943), and 25 unassigned genes were grouped into the MEgrey module, while the final module was generated (Figure 6C,D). The results showed that a total of 5590 genes (93.17%) could be classified into 13 modules (Figure S2). The Turquoise module contained the most genes (943 DEGs), and the MEtan module contained the least number of genes (235 DEGs) (Table S5). The results showed that at least four genes were significantly correlated with heat-stress response in quinoa (*p* < 0.01), including MEbrown (*R* = 0.9, *p* = 6 × 10^−5^), MEgreenyellow (*R* = 0.86, *p* = 3 × 10^−4^), MEgreen (*R* = 0.82, *p* = 0.001) and MEyellow (*R* = 0.8, *p* = 0.002). In addition, the characteristic genes of four modules were negatively correlated with the heat-stress response in quinoa, with MEblue (*R* = −0.96, *p* = 9 × 10^−7^), MEturquoise (*R* = −0.94, *p* = 4 × 10^−6^), MEtan (*R* = −0.89, *p* = 1 × 10^−4^) and MEpink (*R* = −0.85, *p* = 4 × 10^−4^) (Figure 6D). Meanwhile, the MEbrown module eigengene exhibited a strong correlation with the 24 h heat treatment time point (Figure S3).

### 3.8. Analysis of Core Transcriptional Regulators in Response to Heat Stress

**Figure 7:**
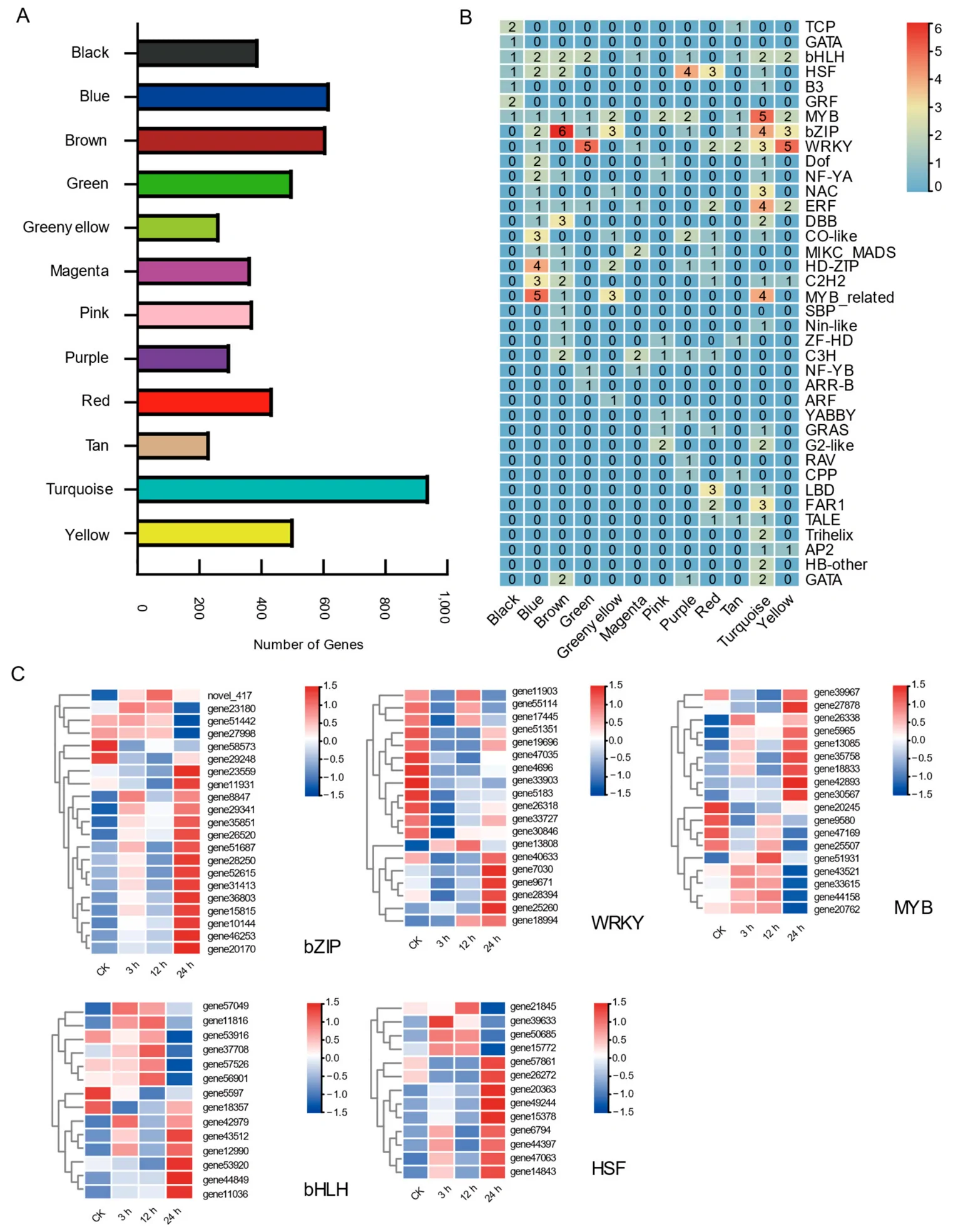
Correlation analysis of gene co-expression modules and transcription factor families. (**A**) Histogram of the number of genes in the co-expression modules with different colors, where the abscissa is the number of genes, showing the difference in the size of each module; (**B**) number of specific transcription factor families in the co-expression module, where the row corresponds to the co-expression module, the color shade and value indicate the enrichment degree of transcription factors in the module (the color scale is from 0 to 6), and the column represents the transcription factor family. The gene composition of the co-expression modules and the enrichment characteristics of transcription factor families in each module are presented; (**C**) the expression patterns of members of five transcription factor families (bZIP, WRKY, MYB, bHLH, HSF) at different stages of heat stress. Each sub-graph corresponds to a transcription factor family, where the rows are marked with specific gene numbers, the columns represent time points, the color shade (red–blue) and the color scale (−1.5 to 1.5) represent the relative changes in gene expression levels, and the tree structure on the left is the hierarchical clustering results of genes. This panel displays the expression pattern differences and clustering characteristics of different families of transcription factors in the time series.

Gene sets with highly specific expression patterns are often closely related to the corresponding phenotypes. In order to explore the network mechanism of regulating heat- stress response, we identified transcription factors for all modules except the MEgrey module (Figure 7). TFs were identified in all modules, involving 38 different families of TFs (Table S6). Among these modules, the Turquoise module had the largest number of transcription factors (49), although it did not have the highest proportion of TFs (5.2%), while the Purple module had the highest proportion of transcription factors in the module (5.32%). The Magenta module had the fewest number of TFs (8) and the lowest proportion of TFs among the genes in the module (2.17%) (Table S7). Among all the transcription factor families, bZIP has the largest number (21) and the highest percentage of all transcription factors (9.55%). For WRKY and MYB, the number of genes was 19 and 18, respectively (Table S8). Interestingly, some transcription factors existed only in a single module (GATA, GRF, SB, etc.).

A large number of transcription factors were enriched in the three stages of heat stress, among which bZIP, WRKY, MYB, bHLH and HSF were the most abundant and differentially expressed transcription factor families. The expression of 15 members of the bZIP transcription factors increased significantly under heat stress, but only *gene23180* and *gene8847* increased significantly in the early stage (3 h), while *gene58573* and *gene29248* decreased to different degrees in different time stages. The rest of the genes were significantly up-regulated at the late stage (24 h). It is worth noting that 417 novel transcripts of the bZIP transcription factor family were newly identified; these transcripts were significantly up-regulated at 12 h under heat stress and played a major role. Most of the WRKY transcription factors were down-regulated to some extent, especially *gene47035*, *gene4696* and *gene33903*. The up-regulated transcription factors were *gene7030*, *gene9671* and *gene25260*, which mainly played a role at 24 h. On the whole, the number of up-regulated and down-regulated MYB transcription factors was basically the same. The genes with the highest fold changes included four up-regulated genes (*gene26338*, *gene35758*, *gene42893*, and *gene30567*) and four down-regulated genes (*gene45521*, *gene33615*, *gene44158*, and *gene20762*). The bHLH transcription factor family was similar to the MYB family with respect to its stress response profile, as both possessed roughly comparable numbers of up- and down-regulated members upon heat treatment. It is noteworthy that *gene5597* and *gene18357* have higher expression levels in the control group, while their expression levels decrease after heat treatment. Different from the above transcription factor families, HSF transcription factors were up-regulated to different degrees after treatment compared with the control group. While the expression levels of *gene39633* and *gene21845* peaked at 3 h and 12 h, respectively, most of the genes reached their highest expression levels at 24 h.

### 3.9. GO and KEGG Pathway Analysis of DEGs

**Figure 8:**
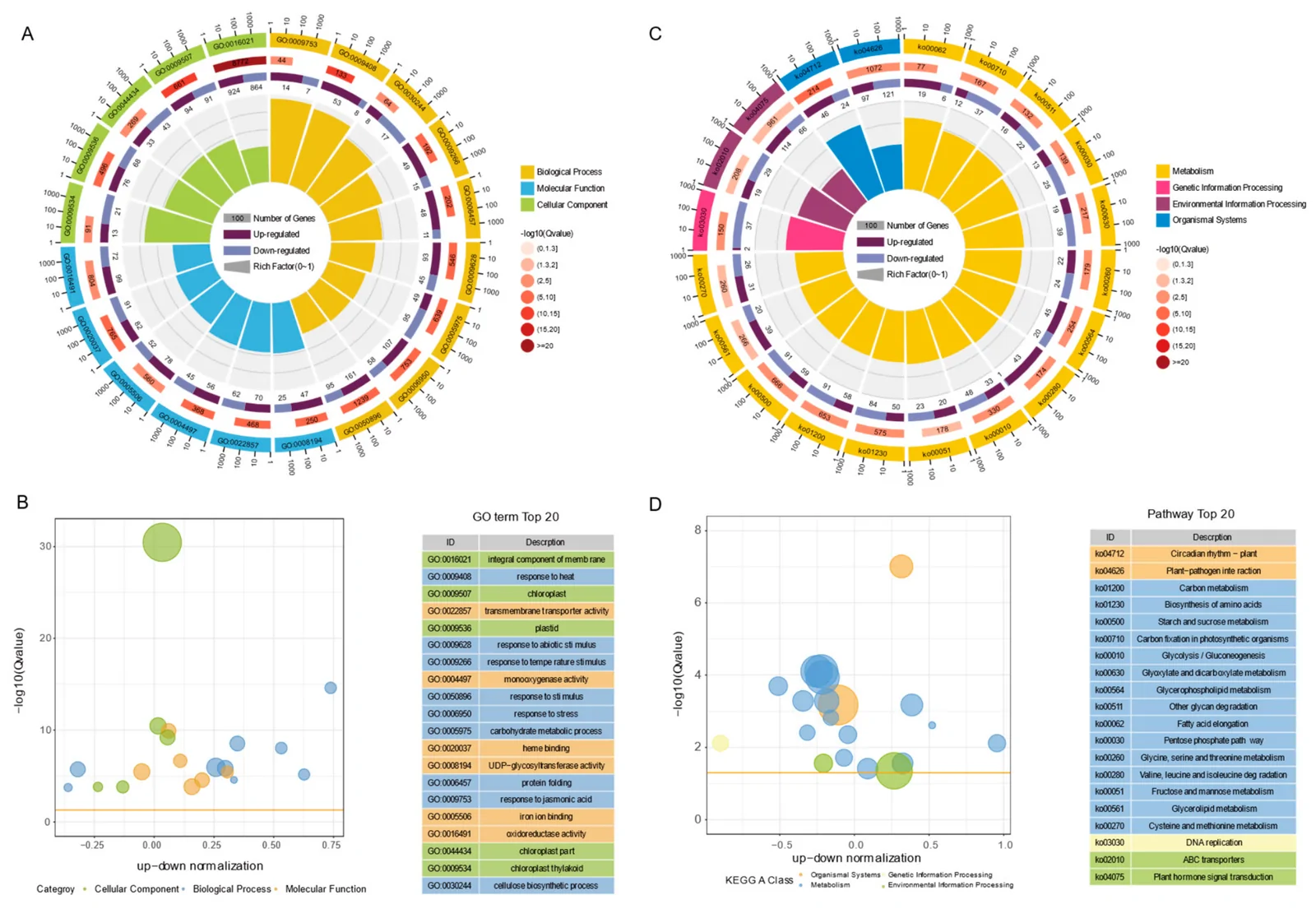
GO and KEGG pathway enrichment analysis maps. (**A**) The circular GO enrichment map, showing the number of enriched genes, the proportion of up-regulated/down-regulated genes, the Rich Factor, the GO term classification and −log10 (*p*-value) from inside to outside. Different colors and numerical values intuitively show the enrichment characteristics of each GO term; (**B**) GO enrichment bubble plot of the top 20 GO terms. The x-axis represents up–down normalization, the y-axis represents −log10(*p*-value), the bubble size indicates the number of genes, and colors denote GO categories, and the yellow line represents the Q value = 0.05; (**C**) a circular KEGG enrichment map, showing the enrichment base factor, the proportion of up-regulated/down-regulated genes, the enrichment factor (Rich Factor), KEGG pathway classification and −log10 (*q*-value) in order from inside to outside; (**D**) KEGG enrichment bubble plot of the top 20 pathways. The x-axis represents up–down normalization, the y-axis represents −log10(*q*-value), the bubble size indicates the number of genes, and colors denote KEGG A-class categories, and the yellow line represents the Q value = 0.05. Different colors and numerical values directly show the enrichment characteristics of each pathway.

To understand the biological scope of transcriptional reprogramming, we performed GO and KEGG enrichment analyses on the identified DEGs (Figure 8). The enrichment profile revealed a hierarchically organized defense response spanning from immediate protein protection to large-scale metabolic and physiological adjustments.

GO enrichment indicated that biological process (BP) terms dominated the enriched categories (9 terms), followed by molecular function (MF, 6 terms) and cellular component (CC, 5 terms). The BP category was overwhelmingly represented by stress-responsive terms, particularly response to heat (53 up-regulated, 8 down-regulated), response to abiotic stimulus (93 up-regulated, 45 down-regulated), and response to temperature stimulus (49 up-regulated, 15 down-regulated). This pronounced activation of heat-shock-related processes aligned with the WGCNA-identified hub genes, which predominantly encode heat shock proteins (HSPs, GO:0006457 protein folding)—confirming that chaperone-mediated protein homeostasis is the primary cellular defense activated by 24 h of heat exposure. At the cellular and molecular levels, the most affected CC terms were integral component of membrane (924 up-regulated, 864 down-regulated), chloroplast (94 up-regulated, 91 down-regulated), and plastid (76 up-regulated, 68 down-regulated). These observations, together with the enriched MF terms such as transmembrane transporter activity (70 up-regulated, 62 down-regulated) and heme binding (82 up-regulated, 91 down-regulated), suggested that prolonged heat stress (24 h) disrupts membrane integrity, plastid function, and electron transport capacity. The near-balanced up/down ratios in membrane and chloroplast terms implied not only damage but also an active remodeling of membrane protein composition to cope with thermal stress. In the 3 h vs. 0 h group, DEGs were more enriched in the GO terms of biological processes, in which metabolic process (GO:0008152) and cellular process (GO:0009987) represented the most abundant genes (Figure S4A). DEGs were also more enriched in biological process GO terms in 12 h vs. 0 h and were the same as the entries enriched in 3 h vs. 0 h (Figure S5A).

To further understand the biological pathway changes involved in heat stress, we performed KEGG enrichment analysis. KEGG pathway analysis extended these insights. Among 307 significantly enriched pathways, the 20 most enriched were categorized into four classes. Metabolism accounted for the largest share (15 pathways), with Carbon metabolism (ko01200, 58 up-regulated, 91 down-regulated), Starch and sucrose metabolism (ko00500, 59 up-regulated, 91 down-regulated), and Glycolysis/gluconeogenesis (ko00010, 33 up-regulated, 48 down-regulated) showing predominantly downregulated gene counts. This indicated a suppression of primary carbon fixation and energy production—a typical metabolic reallocation under sustained heat stress, diverting resources from growth to protection. In the 3 h vs. 0 h group, DEGs were mainly enriched in Plant–pathogen interaction (ko04626), Plant hormone signal transduction (ko04075) and Carbon metabolism (ko01200) (Figure S4B). In the 12 h vs. 0 h group, the KEGG items enriched by DEGs were basically the same as those in the 3 h vs. 0 h, but the proportions were different (Figure S5B). In the 3 h vs. 0 h group, DEGs were mainly enriched in Plant–pathogen interaction (ko04626), Plant hormone signal transduction (ko04075), Carbon metabolism (ko01200), etc. (Figure S4B). In the 12 h vs. 0 h group, the KEGG pathways enriched by DEGs are basically the same as those in the 3 h vs. 0 h, but the proportions were different (Figure S5B).

### 3.10. Molecular Interaction Network Analysis of Quinoa in Response to Heat Stress

**Figure 9:**
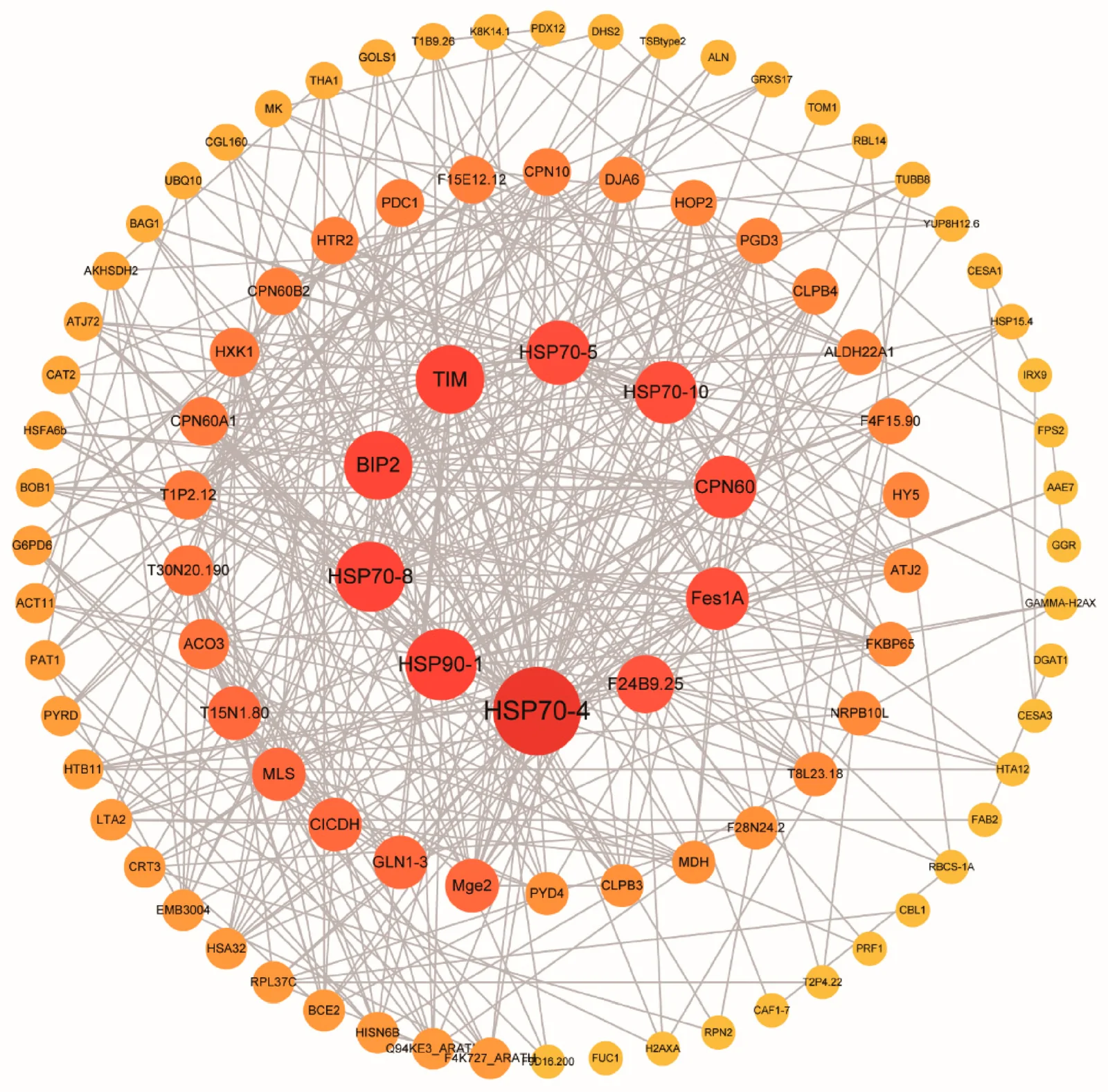
Molecular interaction network analysis of quinoa’s response to heat stress. The figure shows the interaction network dominated by heat shock proteins (HSPs) in quinoa under heat stress. The nodes represent genes/proteins, and the gray lines represent the interactions between molecules (including direct binding, regulation or co-expression association).

Through co-expression network analysis, we further explored the hub genes (key genes) in the strong correlation module and their associations with other genes (Table S9). In Figure 9, the genes in the MEbrown module strongly related to resistance were displayed, with a total of 96 genes included. The inner circle contained 10 pivotal genes (HSP70-4, HSP90-1, HSP70-8, BIP2, TIM, HSP70-5, HSP70-10, CPN60, Fes1A and F24B9.25). Nine of the ten hub genes are chaperone proteins, and seven of them are heat shock proteins (HSP70-4, HSP90-1, HSP70-8, HSP70-5, HSP70-10, BIP2, and CPN60). TIM is a chaperone in the mitochondrial intermembrane space, which is similar to HSP70 but located in the mitochondria, that is responsible for the folding and transport of mitochondrial proteins. Fes1A is an accessory chaperone (nucleotide exchange factor, NEF) of HSP70. F24B9.25, with unknown function, is presumed to be a molecular chaperone related to heat shock proteins, similar to Fes1A. The intensive interaction of the core nodes in the inner circle indicates that they do not play an independent role but form a functionally integrated “protein homeostasis regulatory module”. In summary, the hub genes of this network mainly encode HSPs or other heat-responsive proteins involved in protein folding and thermotolerance. Members of the HSP70 family represented the largest number among these hub genes, which indicates that HSP70 plays a key role in the response to heat stress. Furthermore, HSP70-4 has the most connections, suggesting that it may play the most critical role in heat stress. To verify the reliability of the RNA-sequencing data, we also randomly selected nine DEGs related to our research topic and examined their expression trends at different stress stages. The results showed that the trend of the qRT-PCR data was consistent with the RNA-Seq results (Figure S6), indicating that the quality of the transcriptome data was reliable (primers are listed in Table S10).

## 4. Discussion

Temperature serves as one of the key environmental factors influencing plant growth and development, while also restricting their natural geographical distribution. During the introduction and domestication of crops, investigating the molecular mechanisms underlying plant responses to high temperature is particularly critical. In this study, we performed a comprehensive physiological and transcriptomic analysis of quinoa, analyzing its physiological changes and gene response patterns. Transcriptome analysis revealed that genes involved in the antioxidant defense system and osmotic adjustment substance biosynthesis were responsive to heat stress. Genes associated with the light reaction and photosynthetic carbon assimilation were significantly down-regulated, whereas pathways related to plant hormone signal transduction, amino acid biosynthesis, carbon metabolism, and glycolysis/gluconeogenesis were significantly enriched in the differentially expressed genes. In addition, in combination with the transcriptomic expression profiling, we performed WGCNA to construct an interaction network targeting modular genes significantly related to heat stress. These findings could provide a deeper understanding of the molecular mechanisms of heat stress and identify putative key genes related to heat tolerance, which may provide an important reference for promoting the genetic improvement of heat tolerance in quinoa.

Photosynthesis is one of the most sensitive physiological processes of plants to heat stress, and chlorophyll fluorescence parameters can quickly and sensitively reflect the functional status of the photosynthetic system [38,39]. Fv/Fm, as a core index reflecting the maximum light energy conversion efficiency of PSII reaction centers, has a stable normal value between 0.75 and 0.85. A significant reduction in this parameter provides direct evidence for damage or reversible inactivation of PSII reaction centers [38,40]. In this study, the Fv/Fm of quinoa plants decreased significantly after heat stress, with the largest decrease (25.4%) at 24 h vs. 0 h, which was speculated to be mainly due to the structural damage to the PSII core complex caused by high temperature. On the one hand, high temperature may aggravate the lipid peroxidation of the thylakoid membrane and destroy the structural integrity of the membrane. As a result, the connection between PSII reaction centers and antenna pigment is disordered. On the other hand, high temperature can induce an accelerated degradation rate of the PSII core D1 protein [38,41]. The addition of the nuclear-derived D1 synthesis pathway in *Arabidopsis thaliana* and *Oryza sativa* significantly enhanced the plants’ high-temperature resistance, photosynthetic efficiency, carbon dioxide assimilation rate, biomass, and seed yield [42]. The ΦPSII reflects the light energy conversion efficiency of PSII under actual light conditions, and its down-regulation is directly related to the photosynthetic electron transport efficiency and carbon assimilation capacity of plants [43]. The significant decrease under heat stress was the result of the inhibition of the photosynthetic electron transport chain on the basis of the decrease in Fv/Fm [44]. This indicates that high temperature not only destroys the photochemical activity of PSII reaction centers but also inhibits the function of electron transport carriers on thylakoid membranes, thereby reducing the actual photochemical efficiency [45]. Both qL and qP reflect the open ratio of PSII reaction centers and the efficiency of photosynthetic electron transport, and their significant down-regulation further proved the inhibitory effect of heat stress on the photosynthetic electron transport chain. This indicates that heat stress caused a large number of PSII reaction centers to be closed, which led to a severe impact on photosynthesis. Non-photochemical quenching (NPQ), a key photoprotective mechanism that dissipates excess light energy as heat [46,47], increased sharply at 3 h (by 100% vs. 0 h) but decreased significantly at 24 h (by 30.1%), with no significant change at 12 h. This suggests that heat stress initially enhanced photoprotection, whereas prolonged stress weakened it, potentially reflecting damage to the photosynthetic machinery [41].

In order to avoid the damage caused by heat stress, plants have developed a series of coping strategies, such as the activation of antioxidant enzyme systems and the accumulation of osmotic adjustment substances [48]. In this study, we found that the activities of SOD, POD and CAT in quinoa were significantly increased in a short period of time after heat-stress treatment, which directly reflected the adaptation strategy of quinoa to high-temperature-induced oxidative stress through activating the enzymatic antioxidant systems. In this study, SOD and POD activities increased by 76.7% and 51.4%, respectively, under heat stress, falling within the typical response range of most plants and reflecting a systematic antioxidant defense response, qRT-PCR validation showed that the *CqSOD* and *CqPOD* expression trends were consistent with their corresponding enzyme activities—both peaking at 3 h (78% vs. 76.7%) and 12 h (18% vs. 51.4%), respectively—indicating transcriptional regulation of the antioxidant defense system and validating the reliability of the transcriptome data [49]. However, this also indicates that although the response trend of antioxidant enzymes to temperature may be similar, the specific response patterns are different and are highly dependent on plant organs, growth stages, exposure times and temperatures [50]. Fusion-modified CAT3 enhanced thermotolerance in transgenic tobacco by eliminating excess H_2_O_2_ and mitigating oxidative damage at both the seedling and vegetative growth stages [51]. This indicates that CAT and heat shock proteins may have a certain level of stability to heat stress. Overexpression of *ZmPOD5* in maize significantly increased the levels of SOD and POD under drought stress and significantly decreased the water loss rate, which could also provide a reference direction for water retention of quinoa under heat stress [52].

Plant physiological and metabolic disorders induced by heat stress are the key environmental bottlenecks that restrict plant growth and development and crop yield formation [53]. In the long-term evolutionary process, plants have evolved multi-level adaptive defense strategies to resist high-temperature damage, in which the accumulation of osmotic adjustment substances and the induced expression of functional proteins constitute the core defense pathway. This regulatory model has been verified in many plant species, such as *Brassica rapa*, *Camellia sinensis*, *Sorbus pohuashanensis*, and *Triticum aestivum* [54,55,56,57]. Soluble sugar (SS) accumulation is an important way to enhance the tolerance of plants to heat stress. Its core role is to maintain osmotic balance by increasing the concentration of intracellular solutes and to reduce cell dehydration and plasma membrane damage caused by high-temperature stress [58]. For example, high-temperature acclimation before anthesis can significantly increase the content of soluble sugars such as fructan in wheat stems, alleviate the inhibition of high temperature on the activities of enzymes related to sugar synthesis, and then promote the transport of dry matter to grains after anthesis, alleviating the adverse effects of high temperature on 1000-grain weight [59]. Proline is an amino acid with known osmoprotectant and antioxidant properties [60]. Proline accumulated significantly under heat stress, suggesting that it may act with SOD and POD in ROS scavenging, although its direct functional role was not assessed. The comprehensive role of soluble proteins in heat stress defense cannot be ignored, and the core of its function lies in the superposition of multiple effects of “osmotic adjustment + molecular chaperone protection + antioxidant clearance” [61]. Soluble proteins can also directly play a role in scavenging reactive oxygen species (ROS) and cooperate with proline to build a defense network for plants to cope with adversity [62]. More importantly, HSPs contained in soluble proteins have typical molecular chaperone activity, which can stabilize the spatial structure of enzyme proteins at high temperature and prevent the occurrence of misfolding and aggregation [61]. For example, the expression of the *HSP21* gene in *Arabidopsis thaliana* was significantly up-regulated and maintained at a high level after high-temperature acclimation, and the protein enhanced the survival rate and biomass accumulation of plants under heat stress by protecting photosystem II from oxidative damage [63].

In order to further understand the response of quinoa to heat stress, we performed WGCNA on DEGs at different stages, and the results showed that transcription factors such as HSF, bHLH, GATA, and bZIP were significantly enriched in the MEbrown module, which was highly related to heat stress. We speculate that these transcription factors may play an important role in regulating the transcription of downstream genes. For instance, HSFs have been shown to directly activate antioxidant enzyme genes such as SOD and POD, and their overexpression enhances SOD/POD activities under heat stress, consistent with our observations [64,65]. In quinoa, HSF transcription factors have also been identified as key regulators in response to high-temperature stress, suggesting a conserved role of HSFs in the abiotic stress responses across plant species [66]. At the same time, bHLH35 has multiple functions in *A. thaliana*, in which it not only plays an important role in plant survival and resistance maintenance under high-temperature stress but also positively regulates the response process of salt stress and improves plant salt tolerance; bHLH TFs contribute to ROS homeostasis and stress tolerance [67,68]. In potato, overexpression of *StGATA2* significantly promoted photosynthesis, inhibited transpiration under heat stress to some extent, and increased the contents of H_2_O_2_, MDA and proline, as well as the activities of CAT, SOD and POD, thus improving its tolerance to heat stress [69]. bZIPs participate in heat-stress signaling and osmoprotectant regulation [70]. Thus, the differential expression of these TF families likely orchestrates the transcriptional activation of both antioxidant and osmoprotective pathways, providing a mechanistic link between the transcriptomic changes and the biochemical phenotypes.

Integrative analysis of GO enrichment, KEGG pathways, and the PPI network consistently pointed to protein quality control as a central theme in the quinoa heat-stress response. GO enrichment showed that “response to heat,” “response to temperature stimulus,” and “protein folding” were among the most significantly enriched biological processes, indicating that maintaining protein homeostasis is a major cellular task under heat stress. At the pathway level, KEGG analysis identified “Protein processing in endoplasmic reticulum” (ko04141) as significantly enriched, with *HSP70* and *HSP90* family genes predominantly up-regulated. The PPI network further identified HSP70 and HSP90 as hub proteins with high connectivity, suggesting that they serve as central regulators coordinating the unfolded protein response. Together, these results indicate that heat stress activates a canonical chaperone-mediated protein quality control mechanism in quinoa in which HSP70 and HSP90 play a pivotal role. In a recent transcriptome study of Chinese cabbage under heat stress, significant enrichment of HSP70 and HSP90 was also observed within the ko04141 pathway [71]. Meanwhile, in *OsHSP70* overexpression lines, the tolerance of rice to high temperature was significantly enhanced, and the starch content of rice grains was increased, while in the mutant materials, the starch and protein contents were decreased [72]. This suggests that heat-stress-related chaperone genes such as *HSP70* and *HSP90* may play an important role in the heat-stress response of quinoa, which provides a direction for future molecular breeding of heat tolerance in quinoa.

## 5. Conclusions

**Figure 10:**
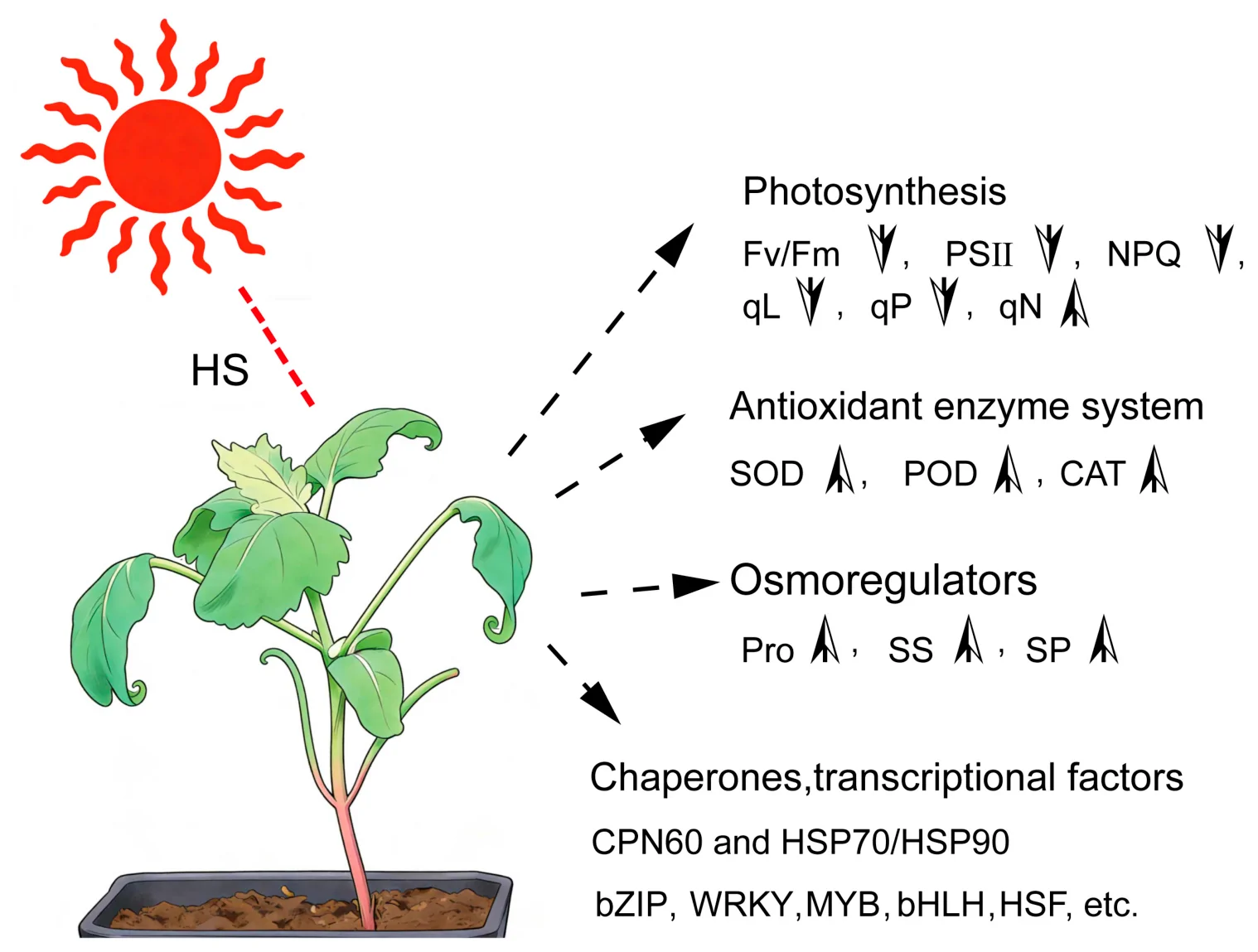
The high-temperature stress response mechanism of quinoa revealed based on transcriptomic and physiological data. Upward arrows indicate upregulation of gene expression and increased enzyme activity, while downward arrows indicate downregulation of gene expression and decreased enzyme activity. Created with Figdraw.

In this study, the activities of antioxidant enzymes and the contents of osmotic adjustment substances in quinoa were determined at different time points after heat-stress treatment. Under heat stress, the activities of antioxidant enzymes such as superoxide dismutase (SOD), peroxidase (POD) and catalase (CAT) were significantly increased, and the contents of osmotic adjustment substances such as proline (Pro), soluble protein (SP) and soluble sugar (SS) were significantly up-regulated. For transcriptome analysis, we focused on the modules highly related to heat stress and constructed the core co-expression network of heat stress using WGCNA. According to the results of WGCNA, we found that the MEbrown module had the highest correlation with heat stress (0.9). Further, a core network visualization analysis of the module with the highest correlation with heat stress was carried out using Cytoscape (version 3.9.1) to obtain the hub genes in the network. A large number of heat shock protein-based chaperones and transcription factor-related co-expressed genes were identified in the core network. Among them, HSP70, HSP90 and CPN60 are located at the core of the core network, which indicates that heat shock proteins may play a key regulatory role in heat stress. Overall, the data obtained in this study will facilitate our understanding of the response network under heat stress and provide valuable resources for in-depth analysis of the HSR regulatory network and future molecular breeding in quinoa and related plant species (Figure 10).

## Figures and Tables

**Table 1:** The overall quality control of the RNA-Seq results.

Sample	Clean Reads (Pairs)	Mapped Reads	UniqMapped Reads	Q20	Q30	GC Content
0 h-1	21,055,520	41,249,290 (97.95%)	37,434,705 (88.90%)	98.51%	95.08%	43.49%
0 h-2	19,483,485	38,136,393 (97.87%)	35,390,852 (90.82%)	98.42%	94.90%	43.49%
0 h-3	23,697,878	46,372,592 (97.84%)	42,295,728 (89.24%)	98.41%	94.83%	43.44%
3 h-1	23,006,208	44,320,656 (96.32%)	40,498,418 (88.02%)	96.64%	91.34%	43.27%
3 h-2	27,480,003	53,599,857 (97.53%)	49,891,728 (90.78%)	98.49%	95.14%	43.58%
3 h-3	20,274,922	39,616,273 (97.70%)	35,732,989 (88.12%)	98.42%	94.89%	43.41%
12 h-1	20,195,596	39,357,921 (97.44%)	36,004,797 (89.14%)	98.20%	94.31%	43.06%
12 h-2	21,178,771	41,298,833 (97.50%)	37,481,410 (88.49%)	98.54%	95.17%	43.21%
12 h-3	19,618,987	38,297,584 (97.60%)	35,449,268 (90.34%)	98.29%	94.56%	43.13%
24 h-1	20,658,836	40,400,302 (97.78%)	37,273,239 (90.21%)	97.94%	93.68%	43.37%
24 h-2	20,170,042	39,514,579 (97.95%)	36,316,868 (90.03%)	98.37%	94.75%	43.43%
24 h-3	20,668,733	40,511,248 (98.00%)	36,540,355 (88.40%)	98.36%	94.72%	43.39%

## Data Availability

Twelve transcriptome datasets were deposited in the NCBI SRA database with accession PRJNA1446775, and the data supporting the findings of this study are available in the article and Supplementary Materials.

## Supplementary Materials

The following supporting information can be downloaded at: https://www.mdpi.com/article/10.3390/biology15191781/s1, Figure S1: Correlation coefficients between three biological replicates at different collection stages after heat stress; Figure S2: Weighted gene co-expression network analysis of DEGs; Figure S3: Module membership vs. gene significance plot; Figure S4: Enrichment analysis of differentially expressed genes at 3 h vs. 0 h; Figure S5: Enrichment analysis of differentially expressed genes at 12 h vs. 0 h; Figure S6: Real-time PCR validation of gene expression levels obtained by RNA-Seq; Table S1: Novel genes predicted by StringTie based on quinoa transcriptome data; Table S2: Novel transcripts predicted by transcriptome sequencing after heat stress in quinoa; Table S3: Transcription factors identified among DEGs at different intervals; Table S4: Expression levels of all genes in the module at different time stages; Table S5: Number of genes in different modules and percentage of all genes; Table S6: Types of transcription factors in different modules; Table S7: Number of transcription factors in different modules as a percentage of total genes; Table S8: Percentage of each transcription factor family; Table S9: Homologous gene alignment using Arabidopsis thaliana as a model species; Table S10: Primer design for qRT-PCR.
