# Supplementary material for: Revealing the Patterns and Key Genes of Heat-Stress Response in Quinoa Through Physiological and Transcriptomic Analyses
Source: Biology (Basel). 2026 Oct 8;15(19):1781. doi: 10.3390/biology15191781 (PMC13664526; doi:10.3390/biology15191781)
Supplement: Supplementary file 1 [file biology-15-01781-s001.zip › Figure S1.pdf]

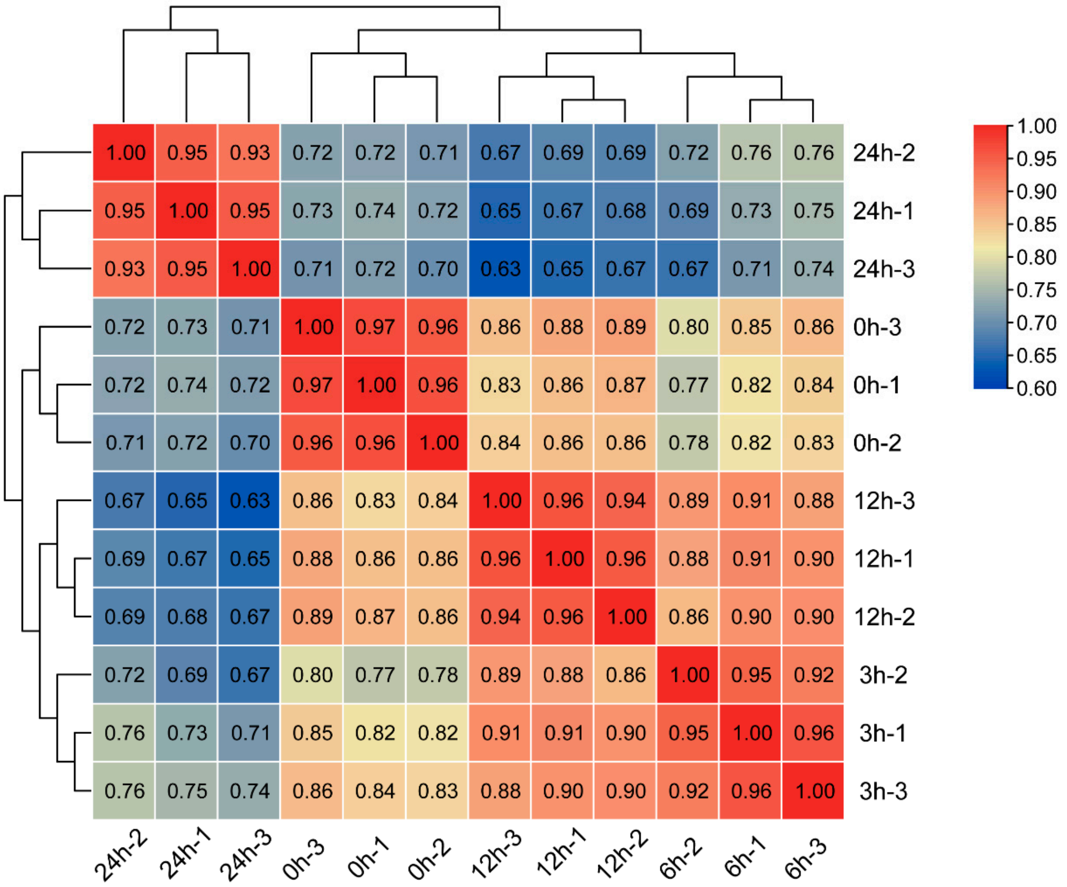

Figure S1 Correlation coefficients between three biological replicates at different collection stages after heat stress. The values were calculated based on the gene expression profile using the cor function of the R language.
