# Supplementary material for: Revealing the Patterns and Key Genes of Heat-Stress Response in Quinoa Through Physiological and Transcriptomic Analyses
Source: Biology (Basel). 2026 Oct 8;15(19):1781. doi: 10.3390/biology15191781 (PMC13664526; doi:10.3390/biology15191781)
Supplement: Supplementary file 1 [file biology-15-01781-s001.zip › Figure.S2.pdf]

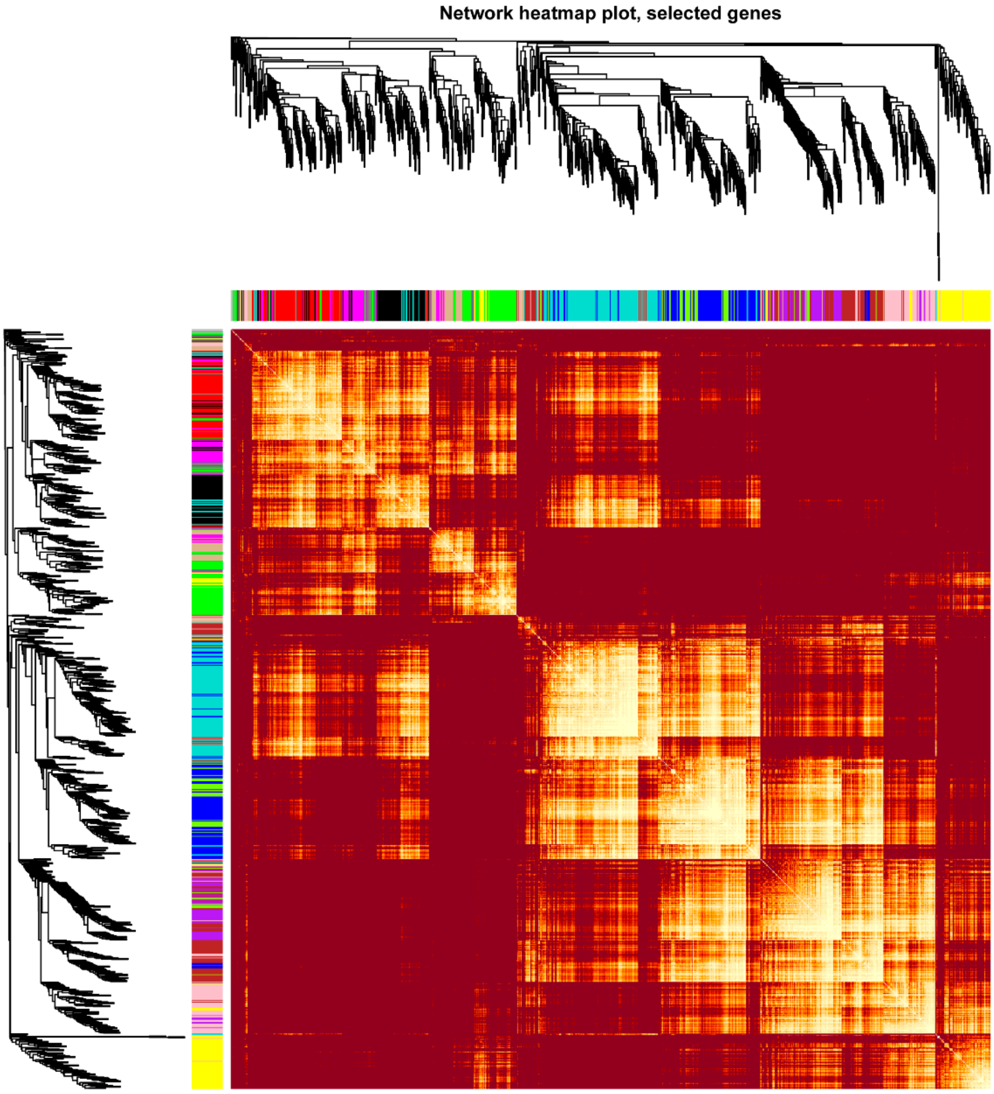


Figure S2 Weighted gene co-expression network analysis of DEGs. 5590 out of 11829 DEGs are grouped into ten gene modules. 393, 623, 612, 503, 375, 235, 301, 439, 943, 368, 267, 506, and 25 in the black, blue, brown, green, pink, tan, purple, red, turquoise, magenta, greenyellow, yellow and grey DEGs were co-expressed , respectively.
