# Supplementary material for: Revealing the Patterns and Key Genes of Heat-Stress Response in Quinoa Through Physiological and Transcriptomic Analyses
Source: Biology (Basel). 2026 Oct 8;15(19):1781. doi: 10.3390/biology15191781 (PMC13664526; doi:10.3390/biology15191781)
Supplement: Supplementary file 1 [file biology-15-01781-s001.zip › Figure.S3.pdf]

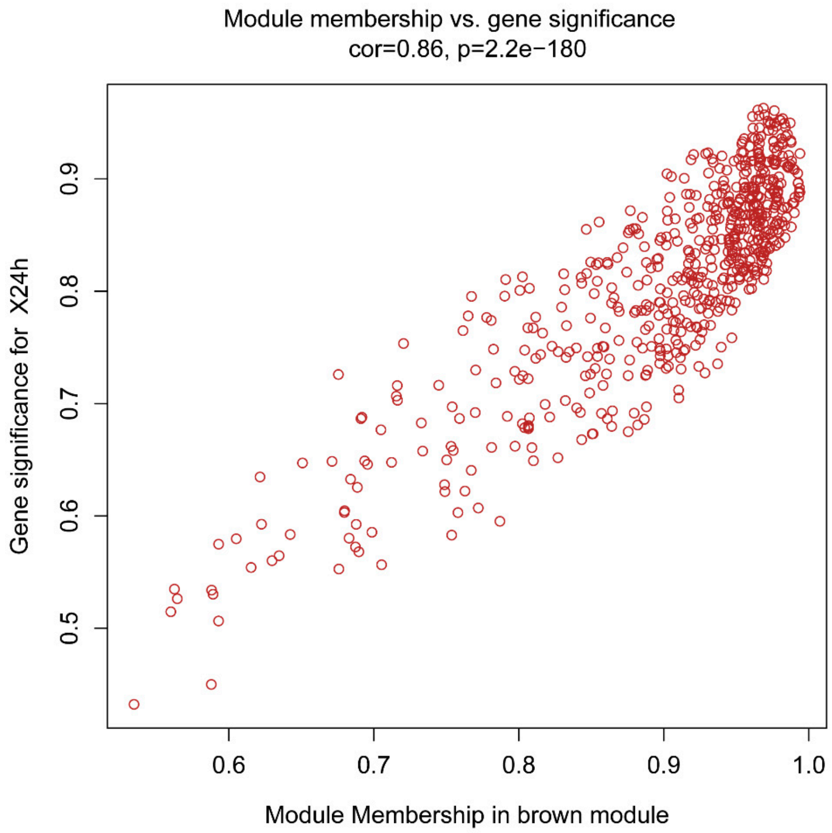

Figure S3 Module membership vs. gene significance plot. The horizontal axis (Module Membership in black module) represents the module membership degree (MM) of each gene in the brown module. The vertical axis (Gene significance for 24 h) represents the significance of association (GS) between each gene and the target time point, with a value of 0-1. The higher the value, the stronger the association between the gene and the trait.
