# Supplementary material for: Revealing the Patterns and Key Genes of Heat-Stress Response in Quinoa Through Physiological and Transcriptomic Analyses
Source: Biology (Basel). 2026 Oct 8;15(19):1781. doi: 10.3390/biology15191781 (PMC13664526; doi:10.3390/biology15191781)
Supplement: Supplementary file 1 [file biology-15-01781-s001.zip › Figure.S4.pdf]

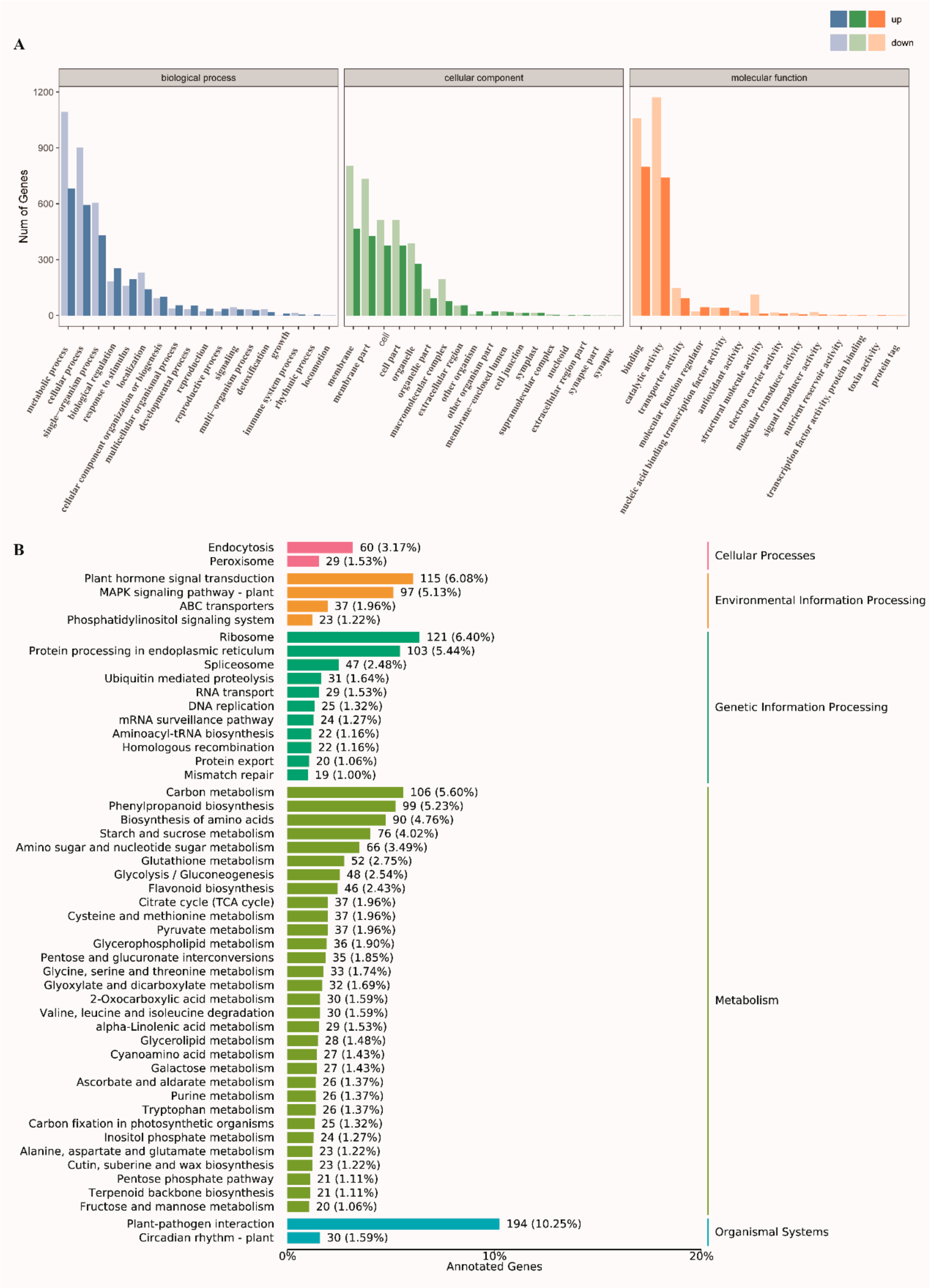

Figure S4 Enrichment analysis of differentially expressed genes at 3 h vs. 0 h. A represents GO items enriched for differentially expressed genes. B represents KEGG items enriched for differentially expressed genes.
