# Supplementary material for: Revealing the Patterns and Key Genes of Heat-Stress Response in Quinoa Through Physiological and Transcriptomic Analyses
Source: Biology (Basel). 2026 Oct 8;15(19):1781. doi: 10.3390/biology15191781 (PMC13664526; doi:10.3390/biology15191781)
Supplement: Supplementary file 1 [file biology-15-01781-s001.zip › Figure.S6.pdf]

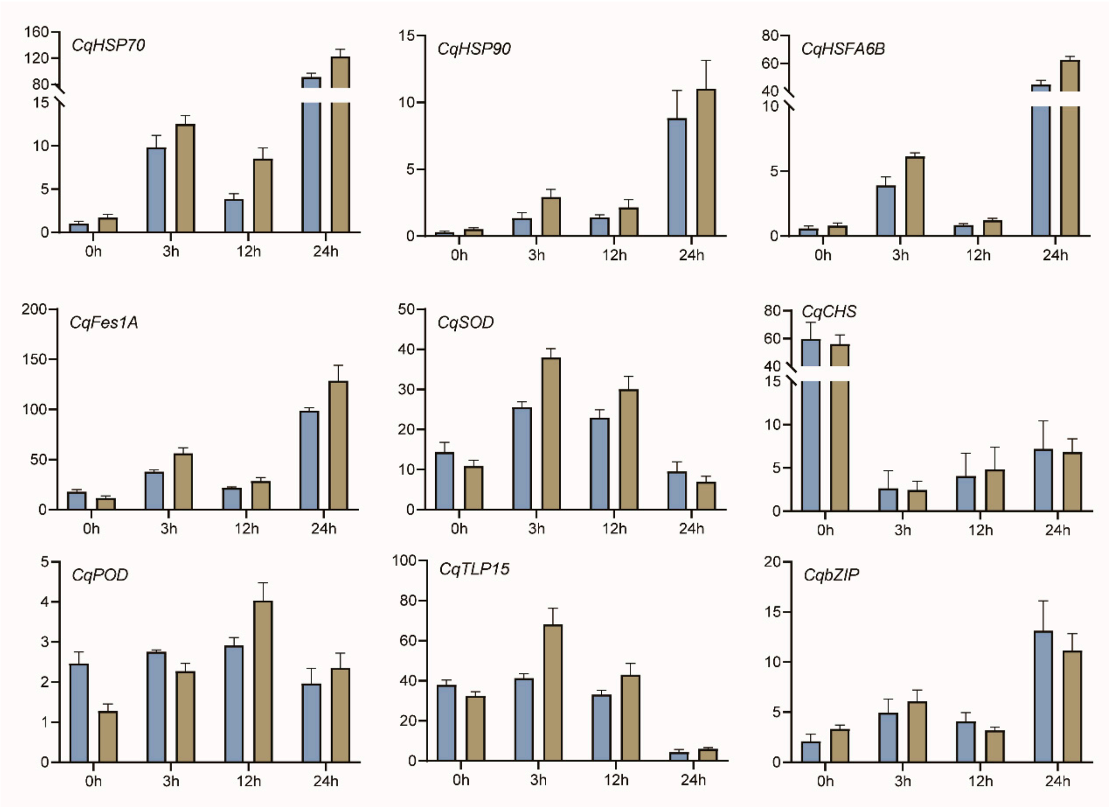

Figure S6 Real-time PCR validation of gene expression levels obtained by RNA-Seq. Light blue and brown bars represent the relative gene expression levels derived from RNA-Seq and real-time PCR, respectively. Real-time PCR values were calculated using the $2^{-\Delta\Delta Ct}$ algorithm, and data are presented as mean ± standard deviation (SD, n=3).
